# Supplementary material for: CRISPR-Cas effector specificity and cleavage site determine phage escape outcomes
Source: PLoS Biol. 2023 Apr 14;21(4):e3002065. doi: 10.1371/journal.pbio.3002065 (PMC10132644; doi:10.1371/journal.pbio.3002065)

Three replicates of gene J cleavage with Cas12a bearing the perfect crRNA. The samples on the left were used in the gel image shown in S1B and S7B Fig. The samples are loaded in the order shown in S1B and S7B Fig.

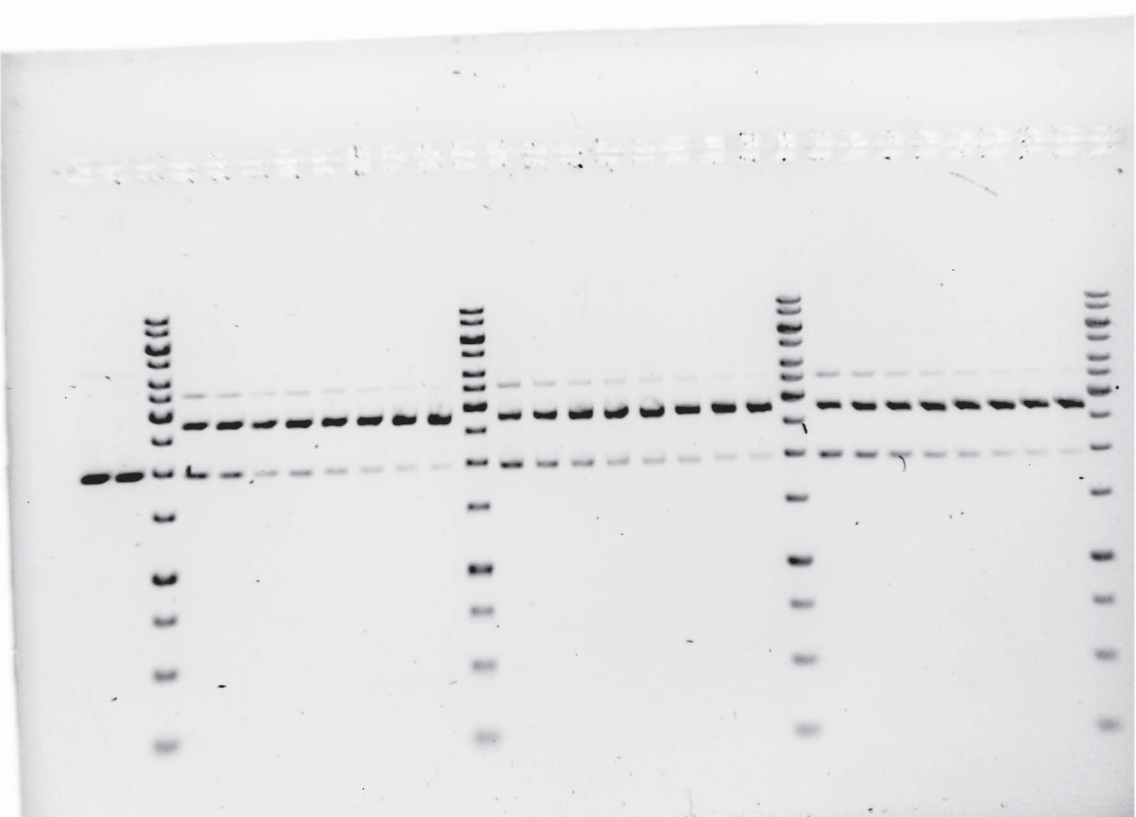

Three replicates of gene J cleavage with Cas12a bearing the MM3 crRNA. The samples on the left were used in the gel image shown in S1B Fig. The samples are loaded in the order shown in S1B Fig.

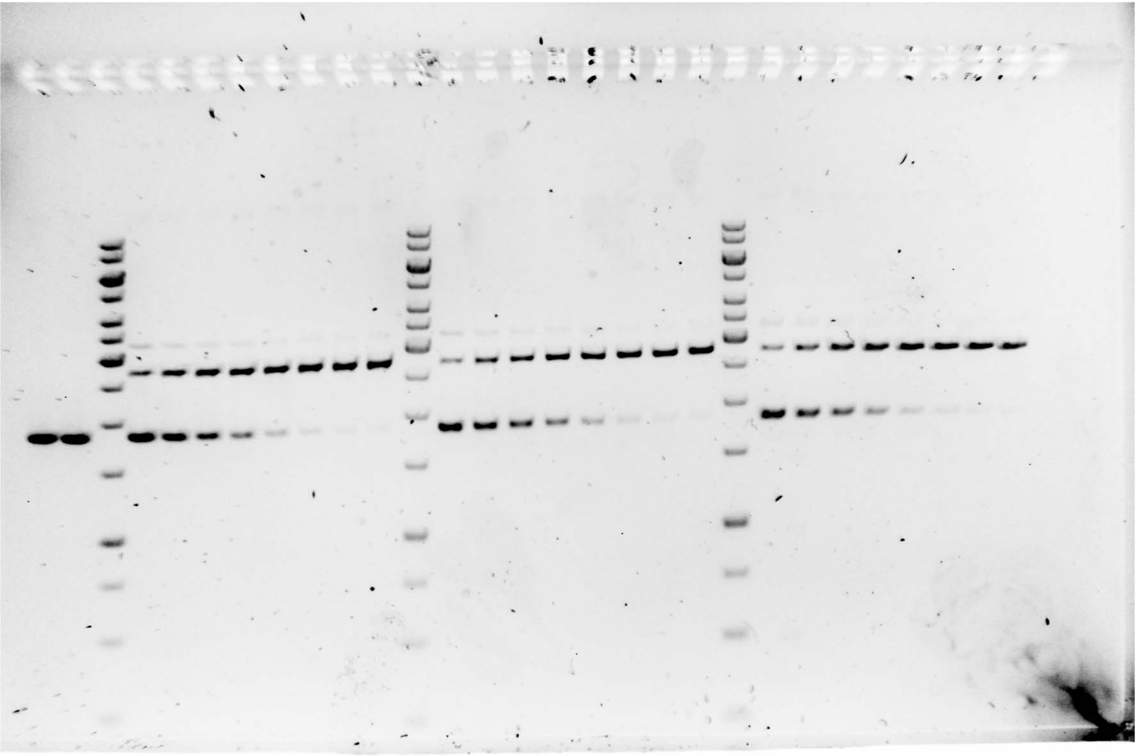

Three replicates of gene J cleavage with Cas12a bearing the MM8 crRNA. The samples on the left were used in the gel image shown in S1B Fig. The samples are loaded in the order shown in S1B Fig.

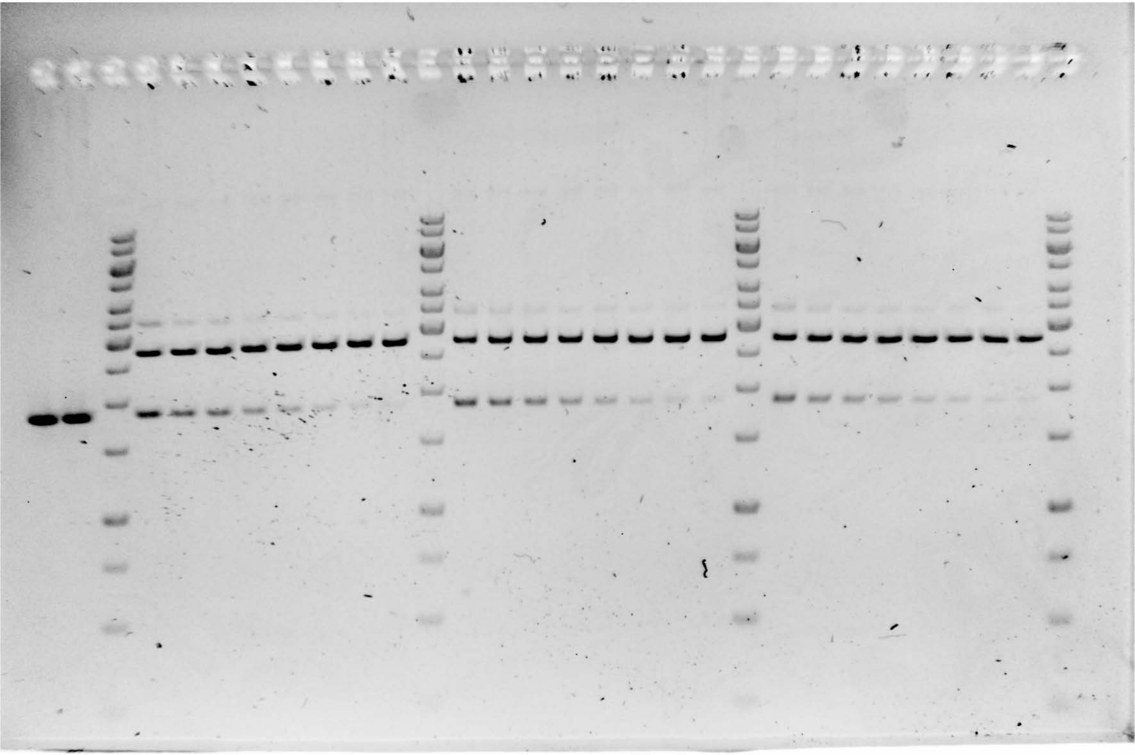

Three replicates of gene J cleavage with Cas12a bearing the MM15 crRNA. The samples on the left were used in the gel image shown in S1B Fig. The samples are loaded in the order shown in S1B Fig.

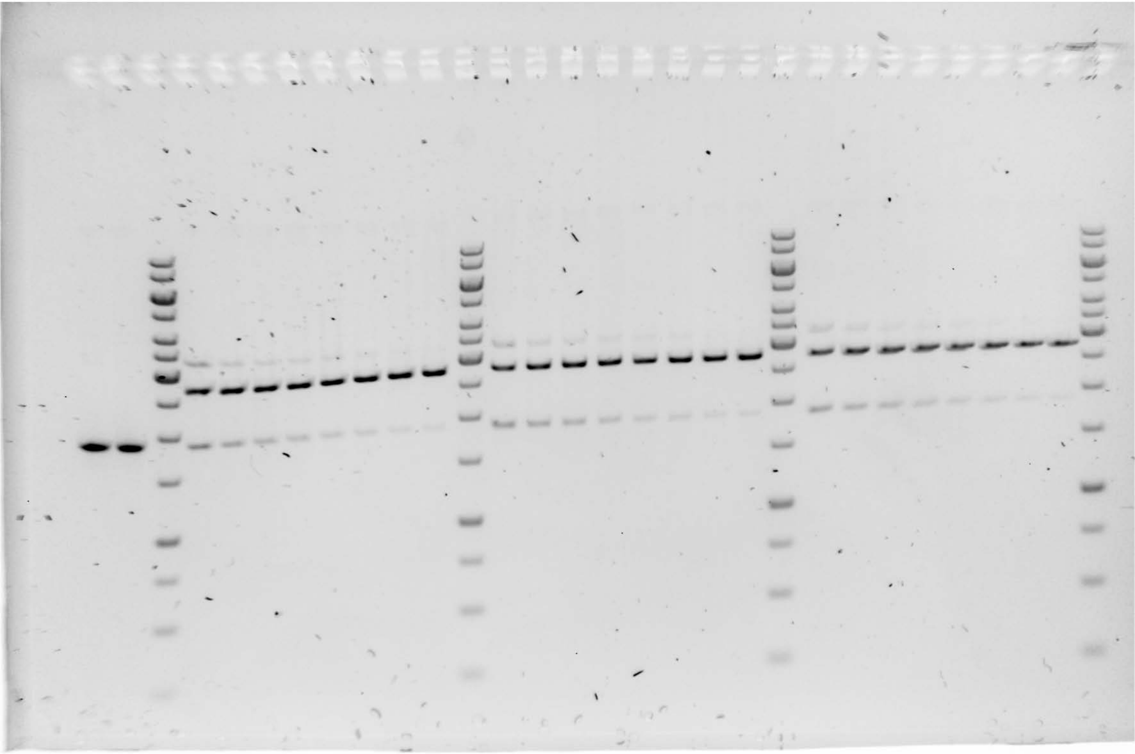

Three replicates of gene J cleavage with Cas12a bearing the MM19 crRNA. The samples on the left were used in the gel image shown in S1B Fig. The samples are loaded in the order shown in S1B Fig.

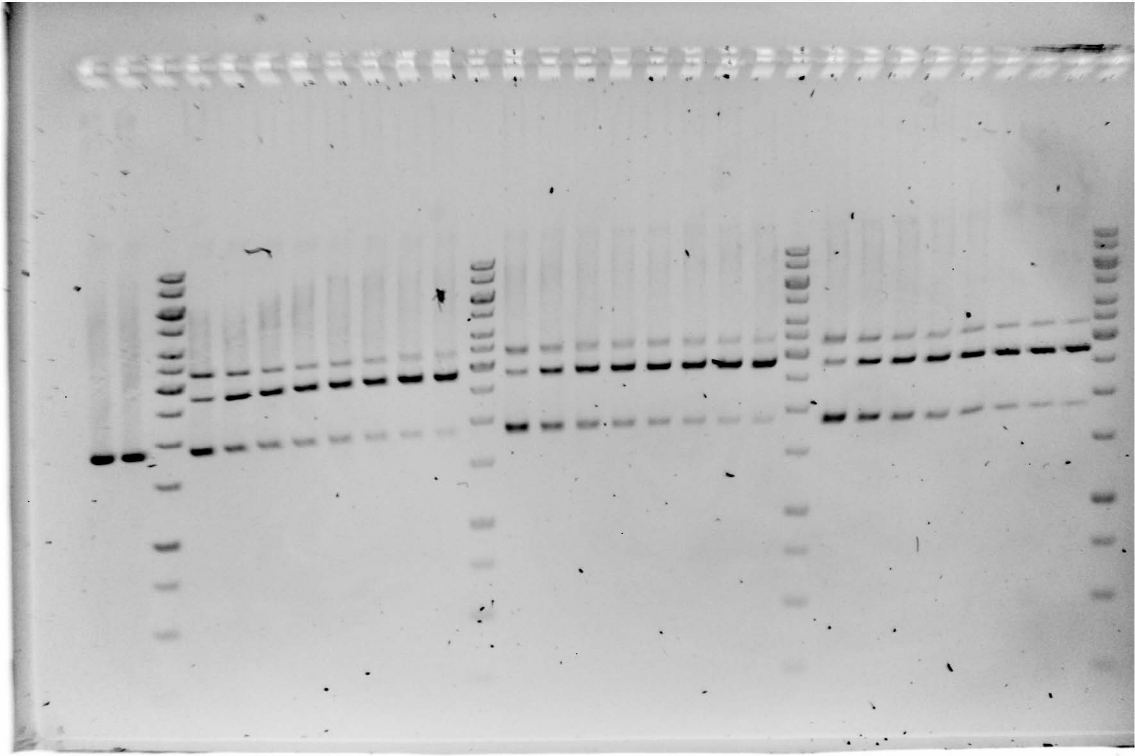

Three replicates of gene L cleavage with Cas12a bearing the perfect crRNA. The samples on the left were used in the gel image shown in S1B and S8B Fig. The samples are loaded in the order shown in S1B and S8B Fig.

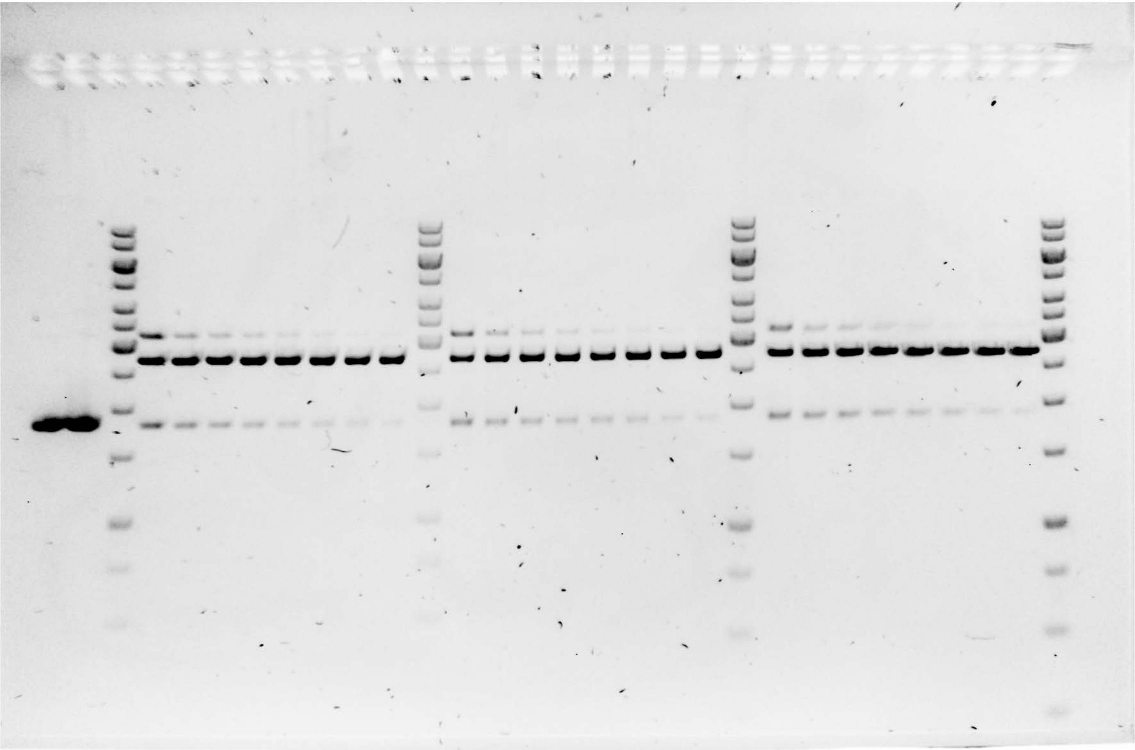

Three replicates of gene L cleavage with Cas12a bearing the MM3 crRNA. The samples on the left were used in the gel image shown in S1B Fig. The samples are loaded in the order shown in S1B Fig.

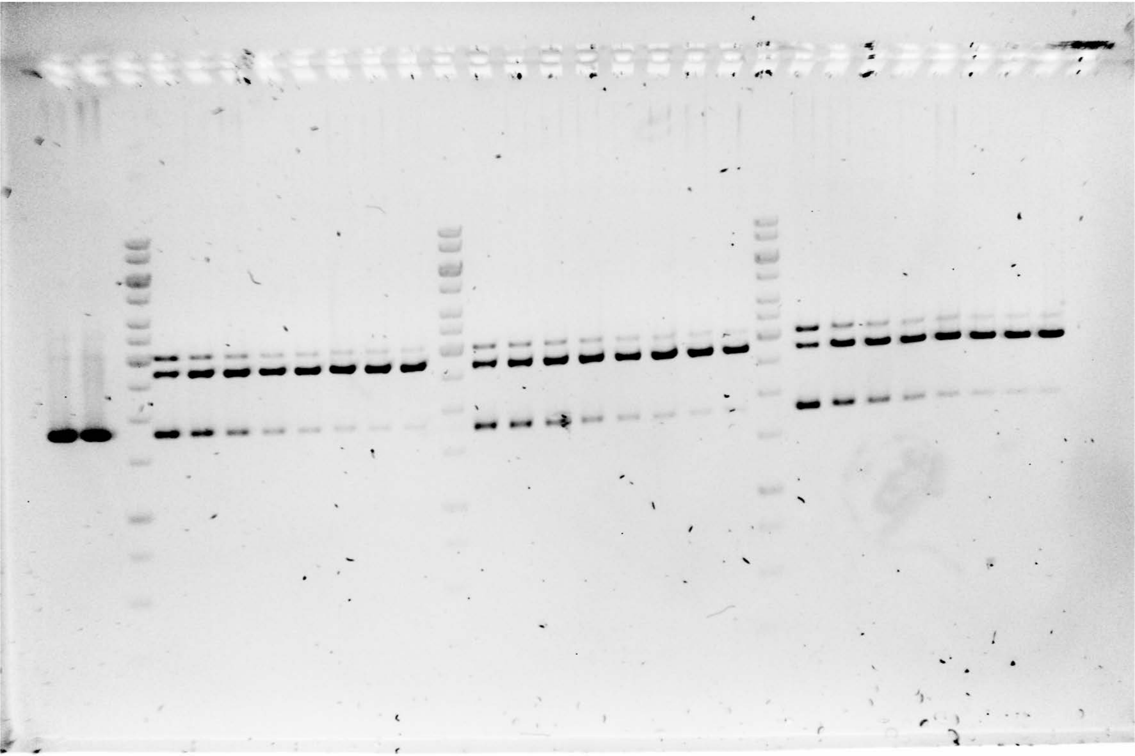

Three replicates of gene L cleavage with Cas12a bearing the MM8 crRNA. The samples on the left were used in the gel image shown in S1B Fig. The samples are loaded in the order shown in S1B Fig.

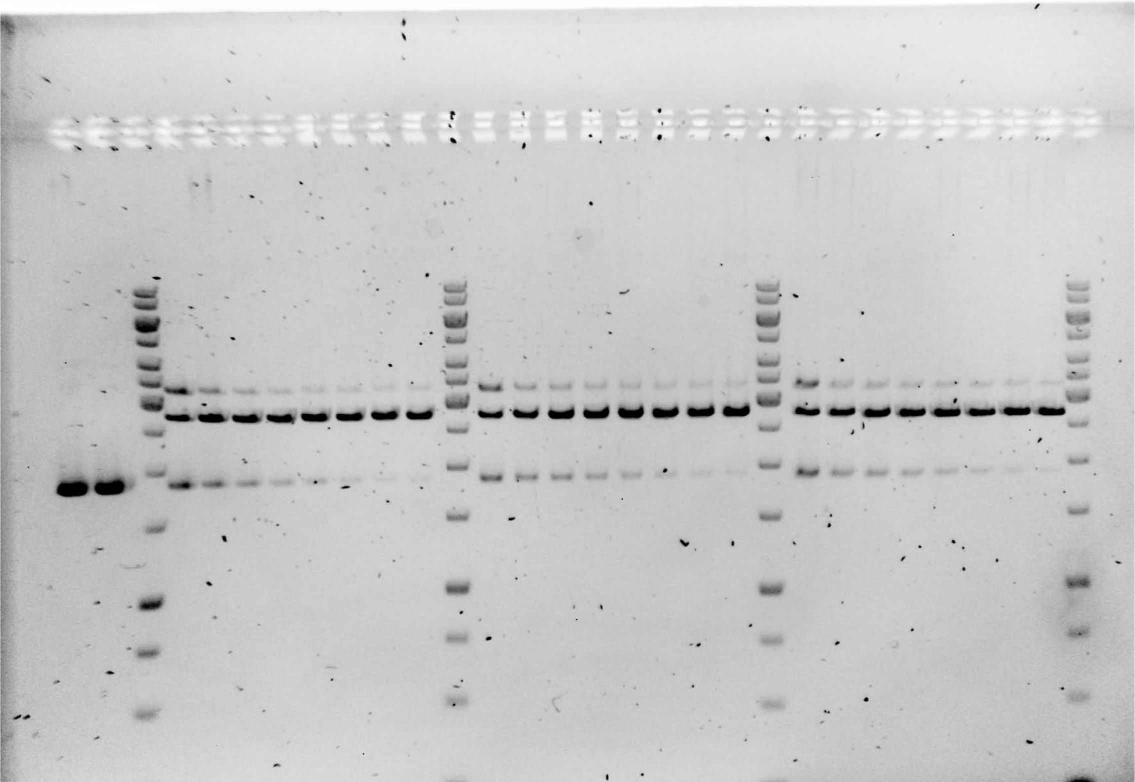

Three replicates of gene L cleavage with Cas12a bearing the MM15 crRNA. The samples on the left were used in the gel images shown in S1B and S8B Fig. The samples are loaded in the order shown in S1B and S8B Fig.

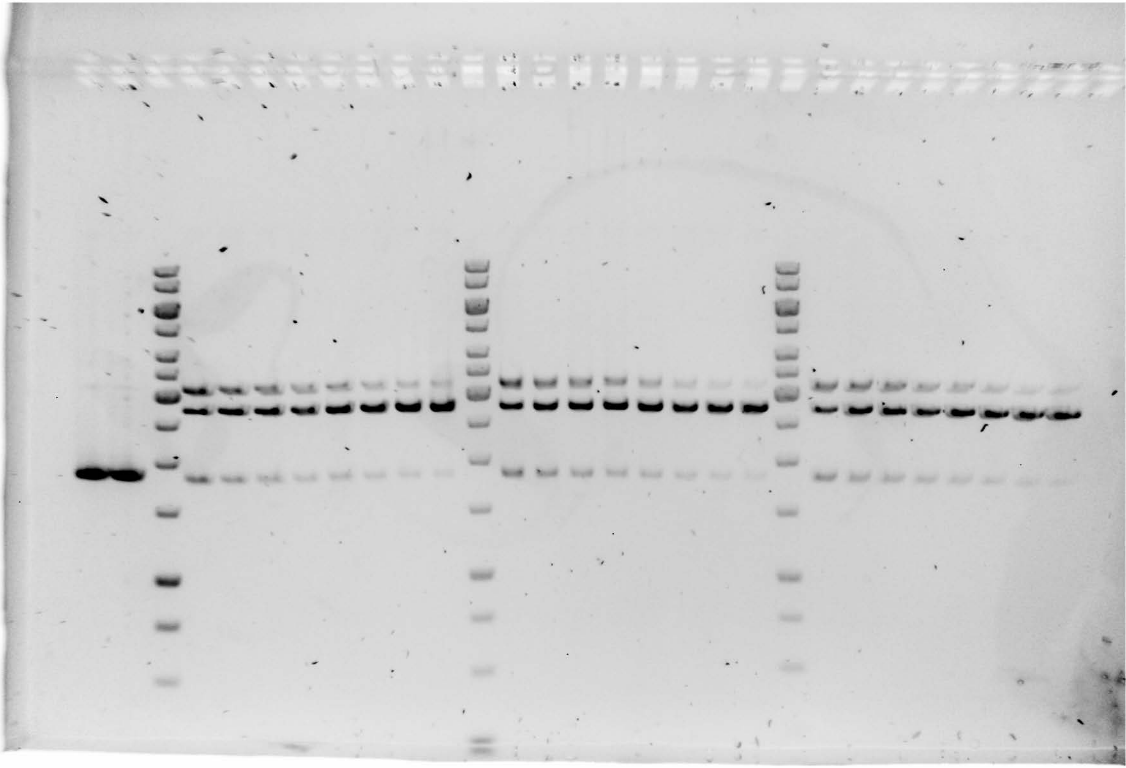

Three replicates of gene L cleavage with Cas12a bearing the MM19 crRNA. The samples on the left were used in the gel image shown in S1B Fig. The samples are loaded in the order shown in S1B Fig.

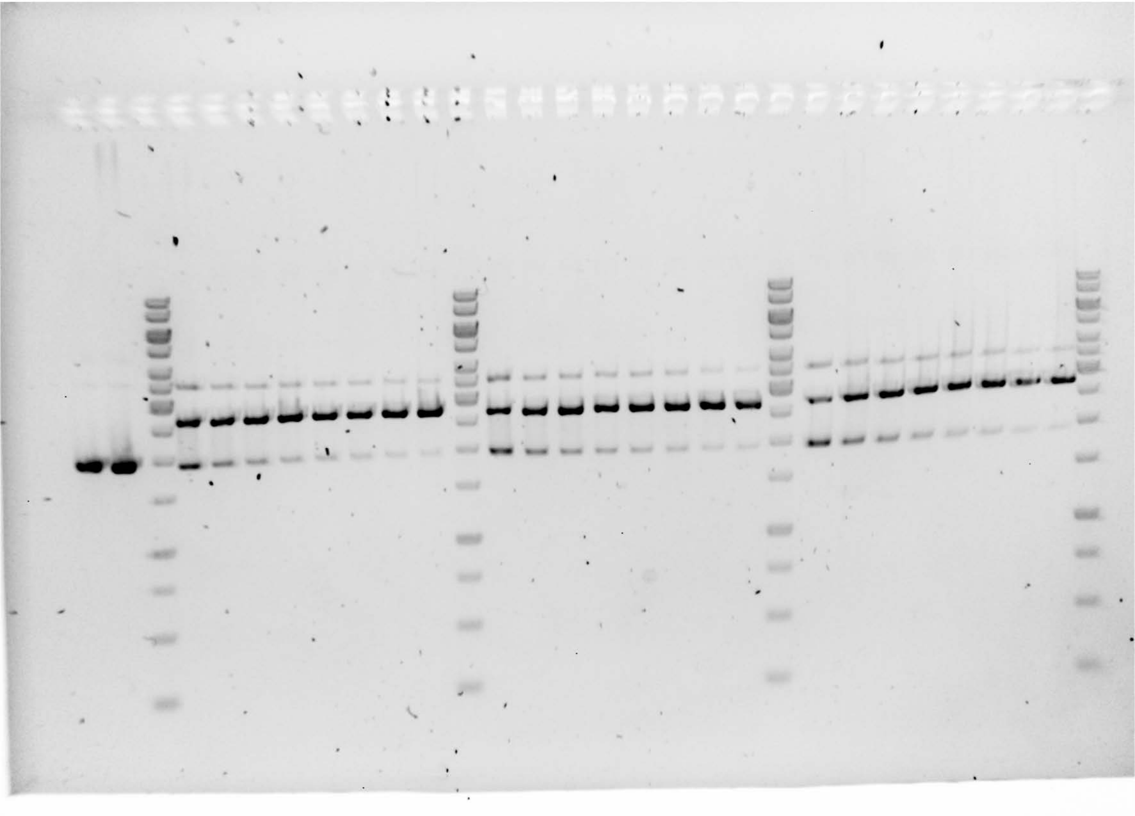

Full gel showing PCR products for amplification of essential and non-essential genes. The samples on the right are shown in the top gel image in S5A Fig. The samples on the left are shown in the bottom gel image in S5A Fig. The samples are ordered as labeled in S5A Fig.

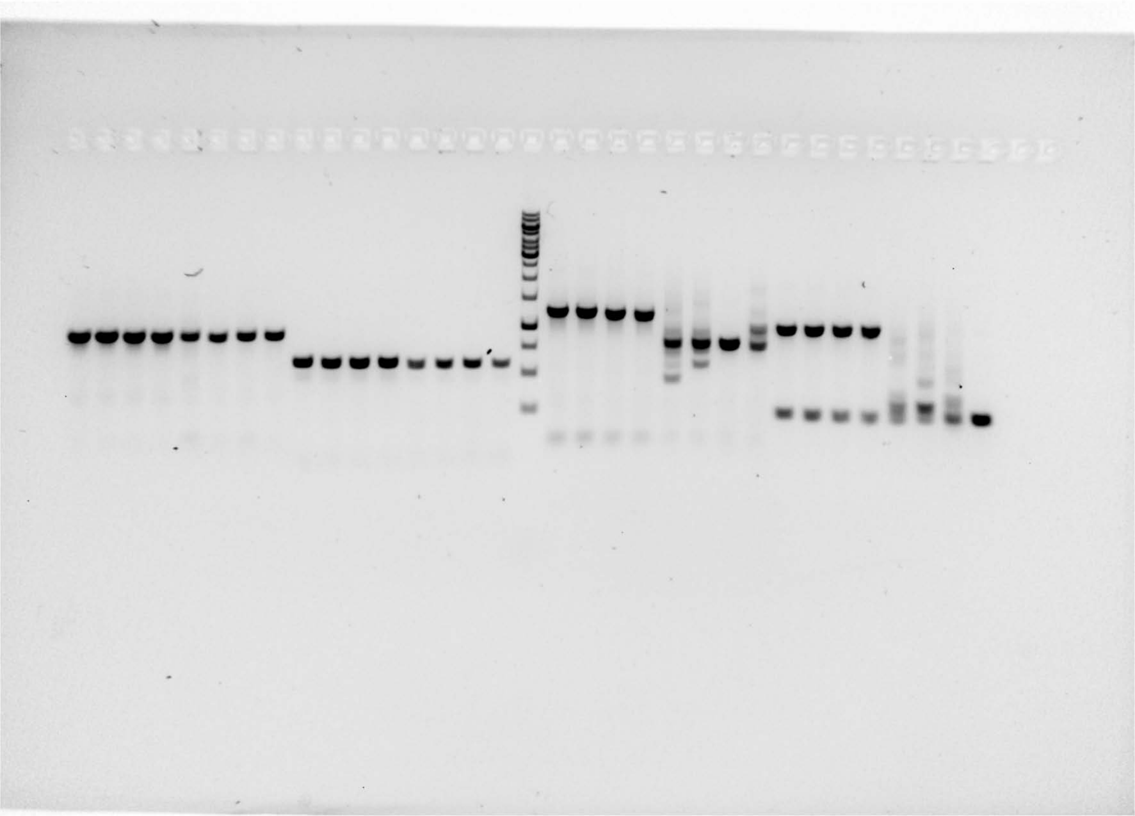

One replicate each of gene J cleavage with Cas12a bearing the 14 (A-C), 15 crRNA (left), the 14 (U-C), 15 crRNA (middle) or the 14 (C-C), 15 crRNA (right.) The samples on the left and middle were used in the gel image shown in S7B Fig. The samples are loaded in the order shown in S7B Fig.

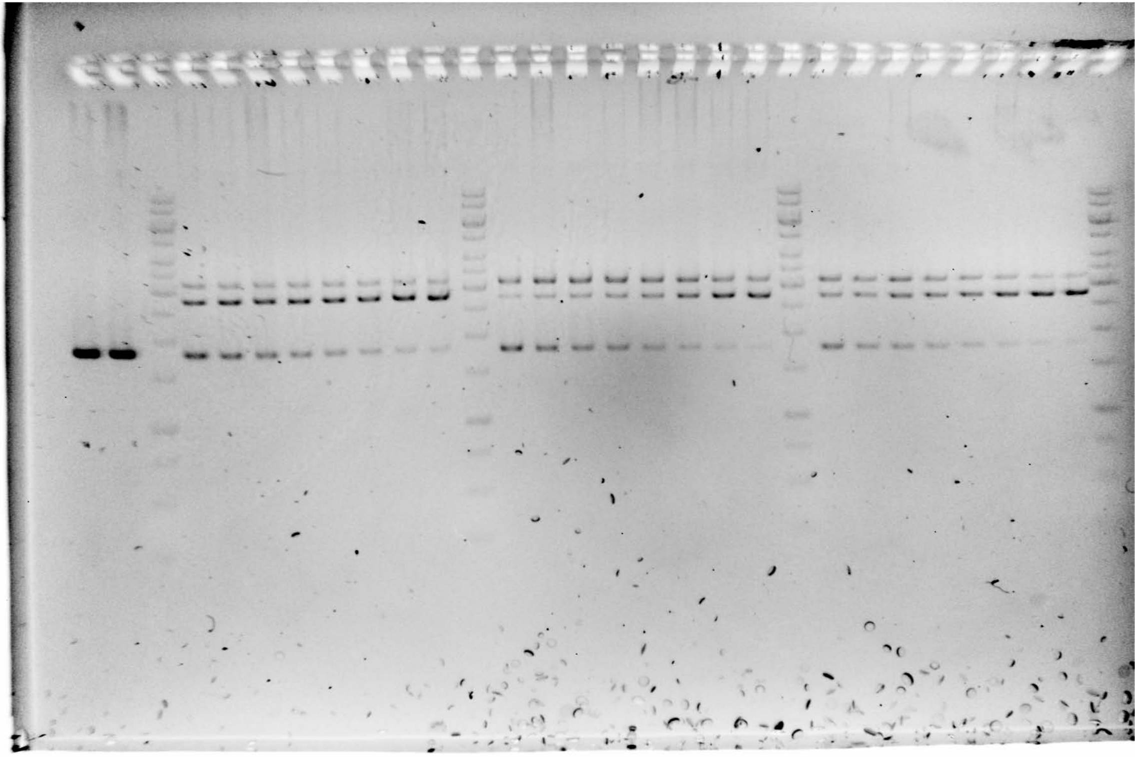

One replicate of gene J cleavage with Cas12a bearing the 14 (U-C), 15 crRNA (left) and two replicates of gene J cleavage with Cas12a bearing the 14 (C-C), 15 crRNA (middle, right). The samples in the middle were used in the gel image shown in S7B Fig. The samples are loaded in the order shown in S7B Fig.

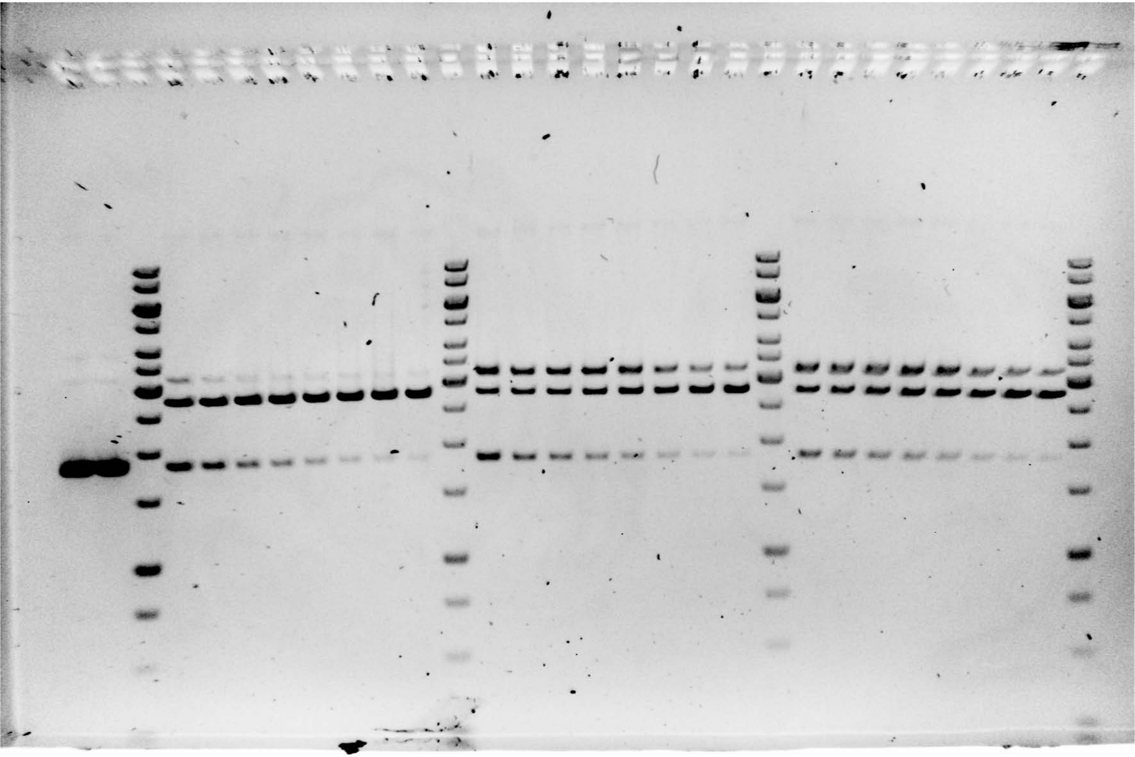

Two replicates of gene J cleavage with Cas12a bearing the 14 (A-C), 15 crRNA (left, middle) and one replicate of gene J cleavage with Cas12a bearing the 14 (U-C), 15 crRNA (right). The samples are loaded in the order shown in S7B Fig.

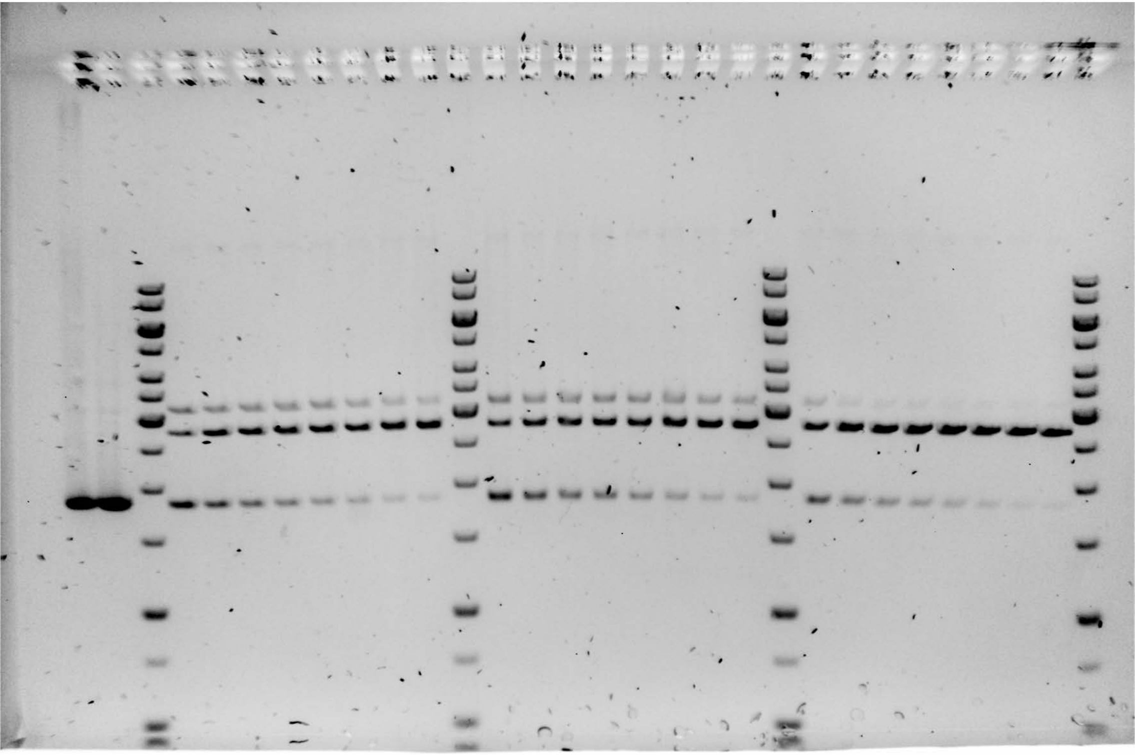

One replicate of gene J cleavage with Cas12a bearing the 16 (A-C), 19 crRNA (right). The samples on the right were used in the gel image shown in S7B Fig. The samples are loaded in the order shown in S7B Fig. The samples on the left were not relevant to this manuscript.

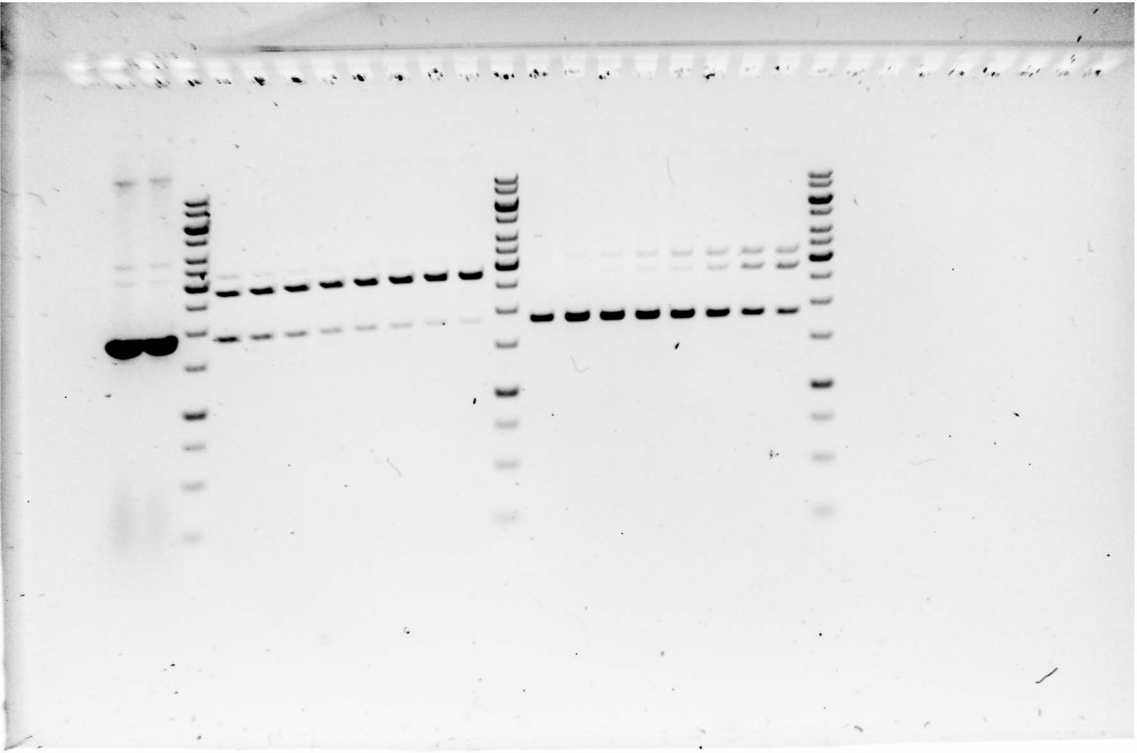

One replicate each of gene J cleavage with Cas12a bearing the 16 (U-C), 19 crRNA (left) or the 16 (C-C), 19 crRNA (right). The samples were used in the gel image shown in S7B Fig. The samples are loaded in the order shown in S7B Fig.

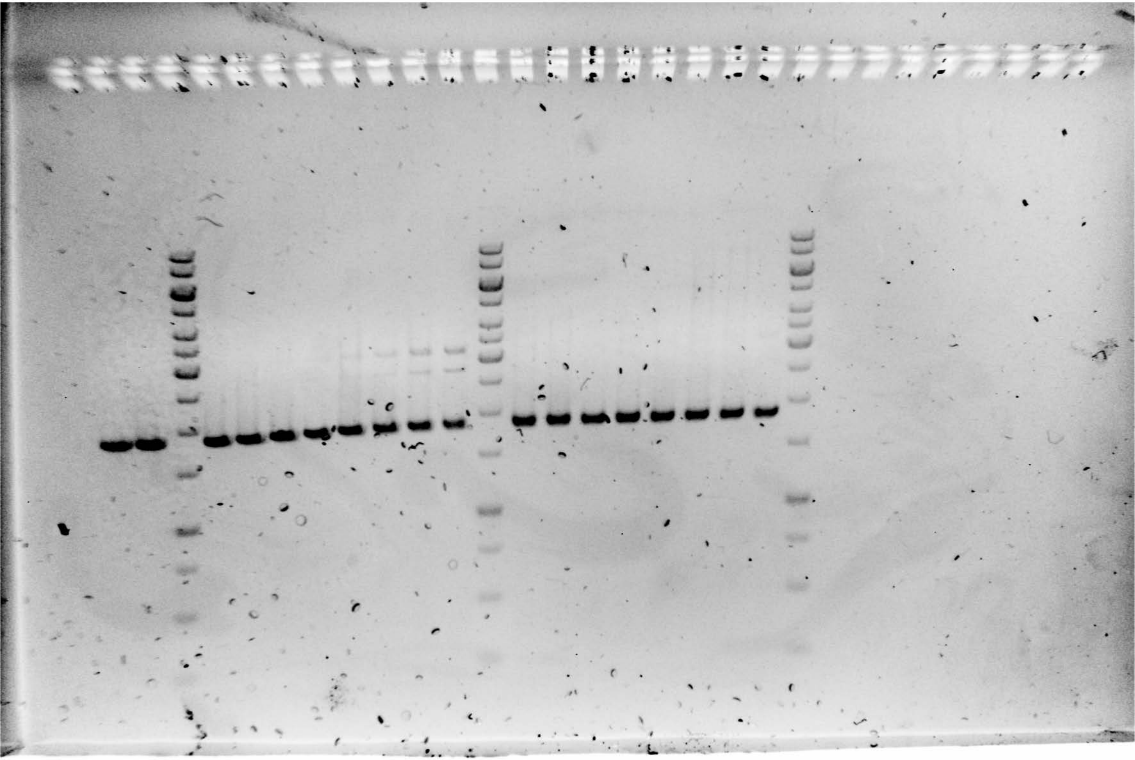

One replicate each of gene J cleavage with Cas12a bearing the 16 (A-C), 19 crRNA (left), the 16 (U-C), 19 crRNA (middle) or the 16 (C-C), 19 crRNA (right). The samples are loaded in the order shown in S7B Fig.

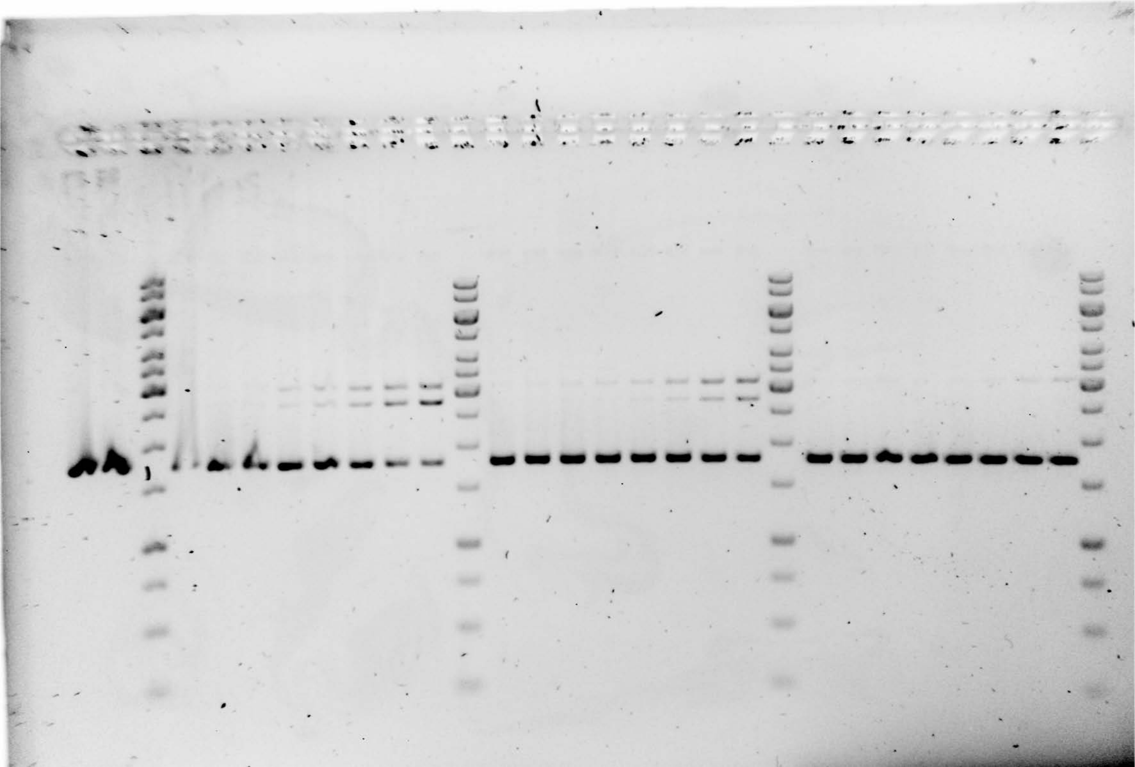

One replicate each of gene J cleavage with Cas12a bearing the 16 (A-C), 19 crRNA (left), the 16 (U-C), 19 crRNA (middle) or the 16 (C-C), 19 crRNA (right). The samples are loaded in the order shown in S7B Fig.

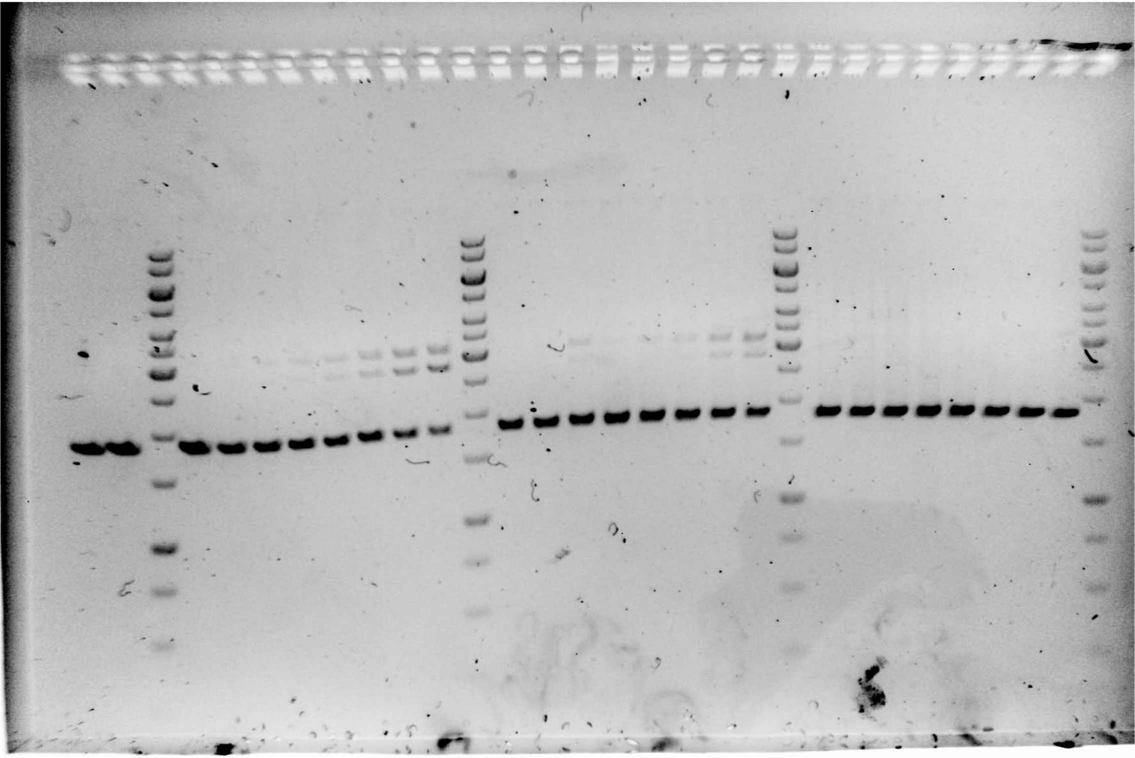

One replicate of A2T gene L cleavage with Cas12a bearing the perfect crRNA (left). The samples on the left were used in the gel image shown in S8B Fig. The samples are loaded in the order shown in S8B Fig. The samples on the right were not relevant to this manuscript.

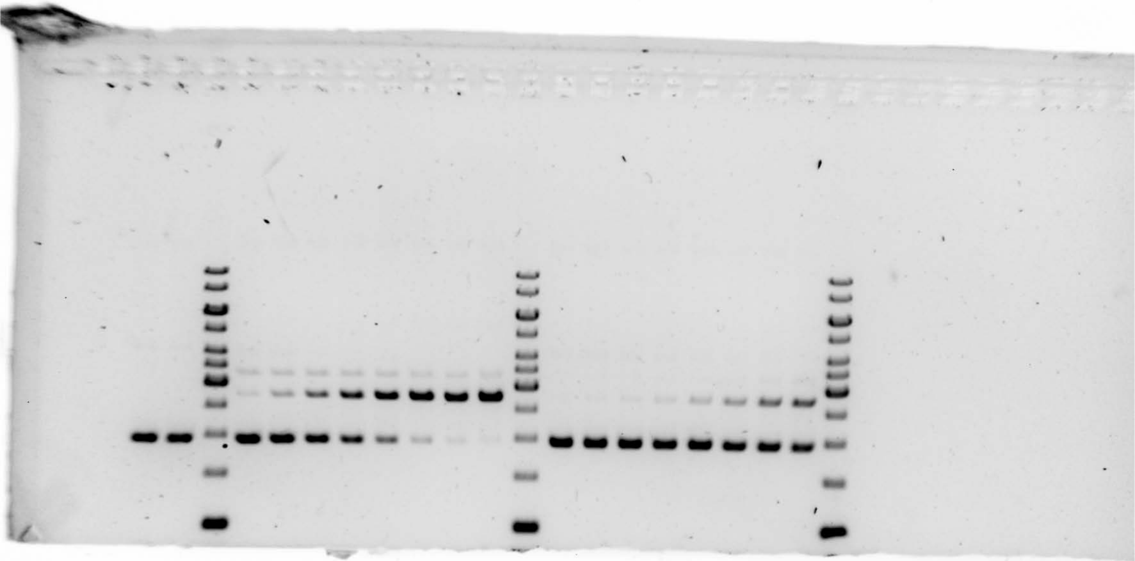

One replicate of A2T gene L cleavage with Cas12a bearing the perfect crRNA (left). The samples are loaded in the order shown in S8B Fig. The samples on the right were not relevant to this manuscript.

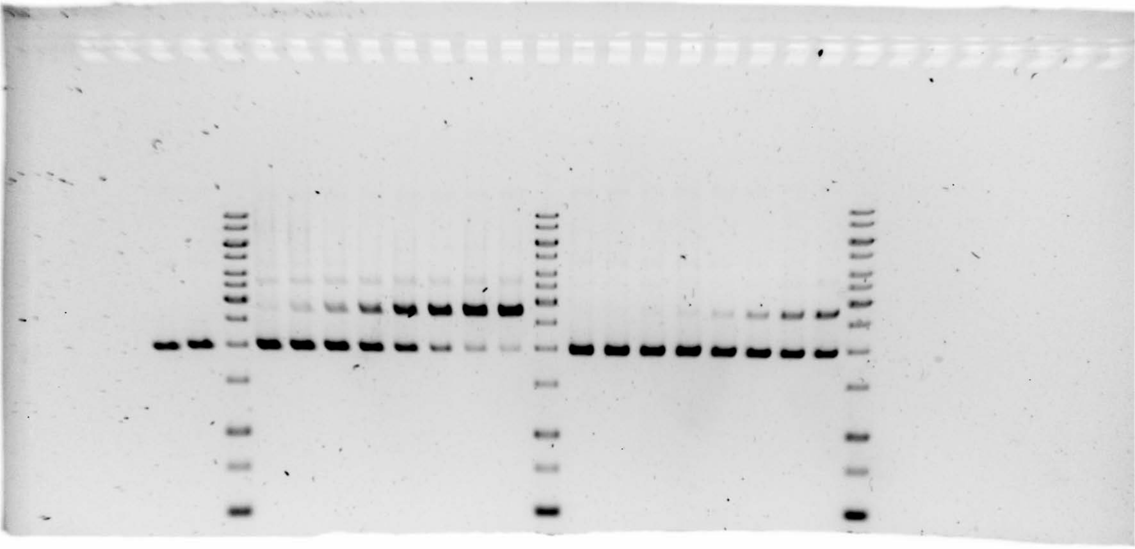

One replicate of A2T gene L cleavage with Cas12a bearing the perfect crRNA (left). The samples are loaded in the order shown in S8B Fig. The samples on the right were not relevant to this manuscript.

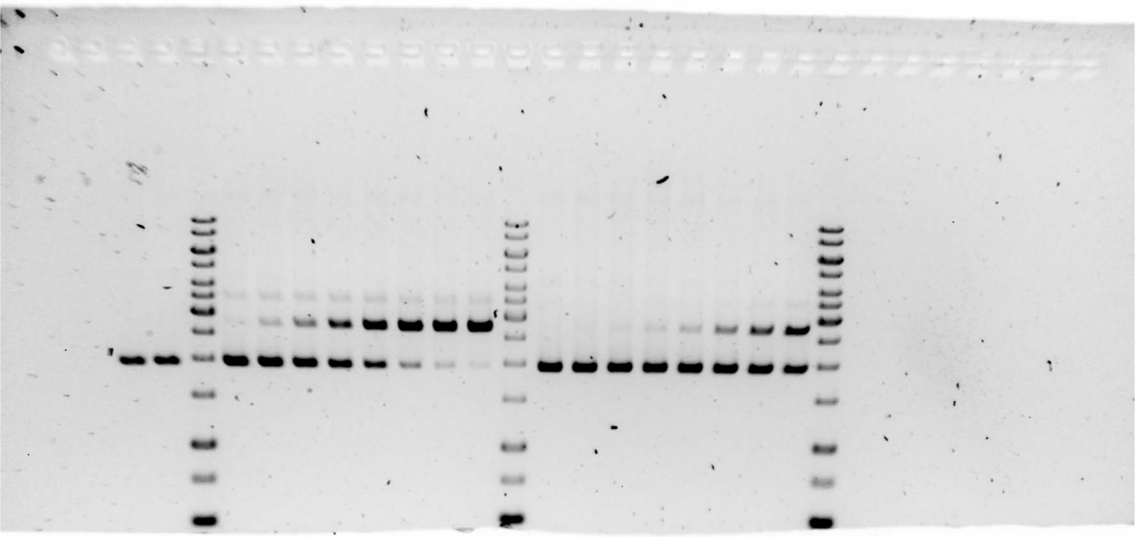

Three replicates of G17T gene L cleavage with Cas12a bearing the perfect crRNA. The samples on the left were used in the gel image shown in S8B Fig. The samples are loaded in the order shown in S8B Fig.

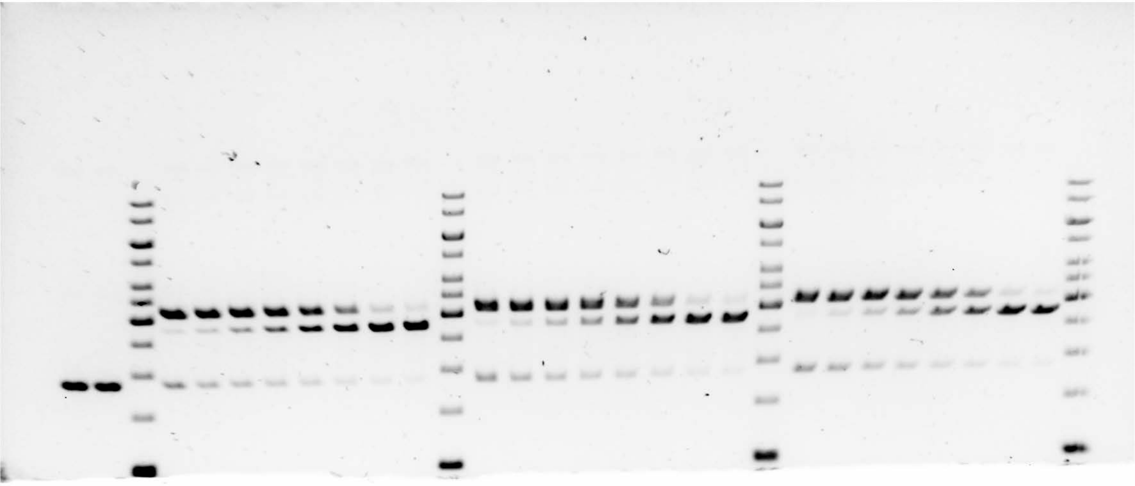

One replicate of A2T gene L cleavage with Cas12a bearing the MM15 crRNA (left). The samples on the left were used in the gel image shown in S8B Fig. The samples are loaded in the order shown in S8B Fig. The samples on the right were not relevant to this manuscript.

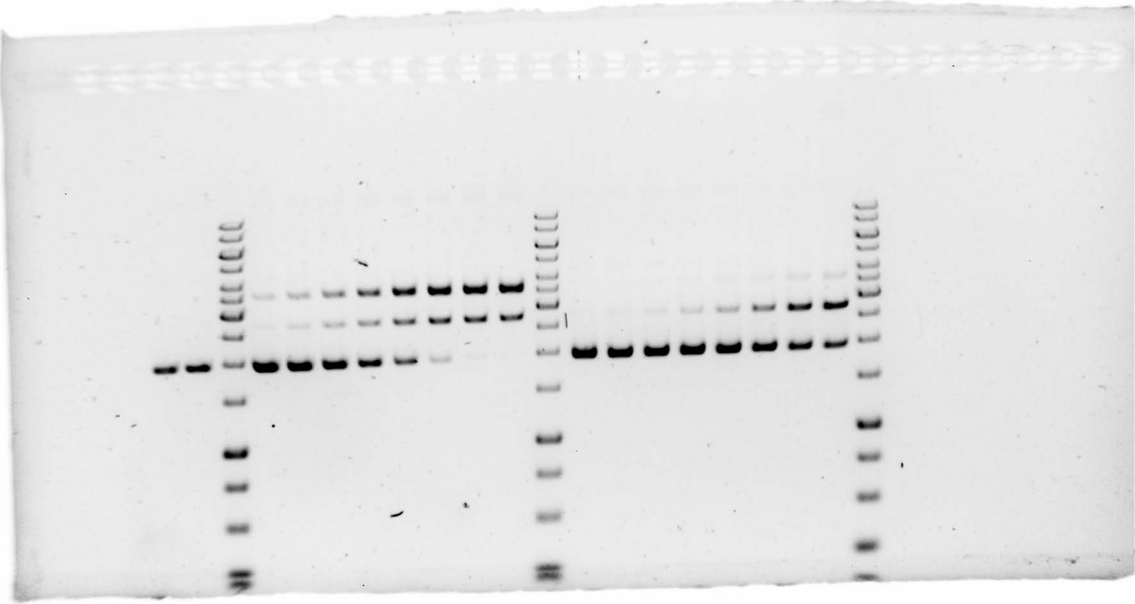

One replicate of A2T gene L cleavage with Cas12a bearing the MM15 crRNA (left). The samples are loaded in the order shown in S8B Fig. The samples on the right were not relevant to this manuscript.

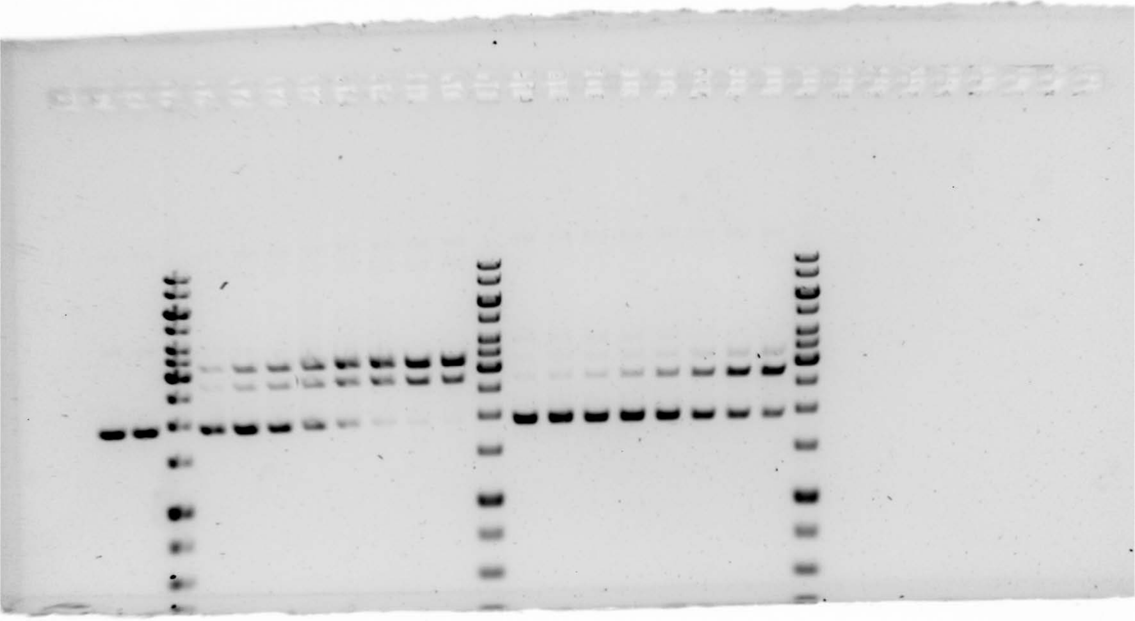

One replicate of A2T gene L cleavage with Cas12a bearing the MM15 crRNA (left). The samples are loaded in the order shown in S8B Fig. The samples on the right were not relevant to this manuscript.

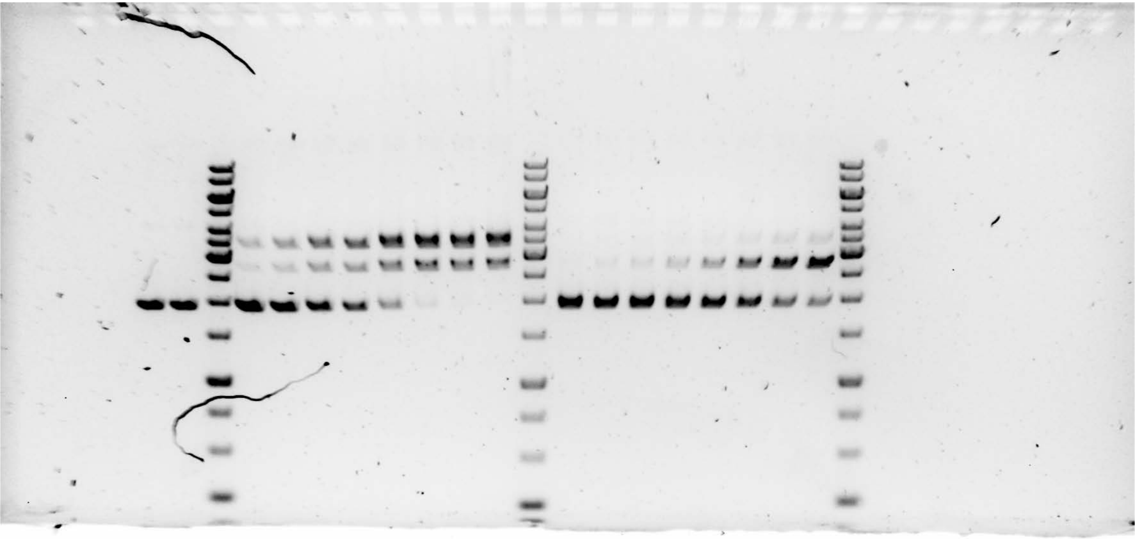

Three replicates of G17T gene L cleavage with Cas12a bearing the MM15 crRNA. The samples on the left were used in the gel image shown in S8B Fig. The samples are loaded in the order shown in S8B Fig.

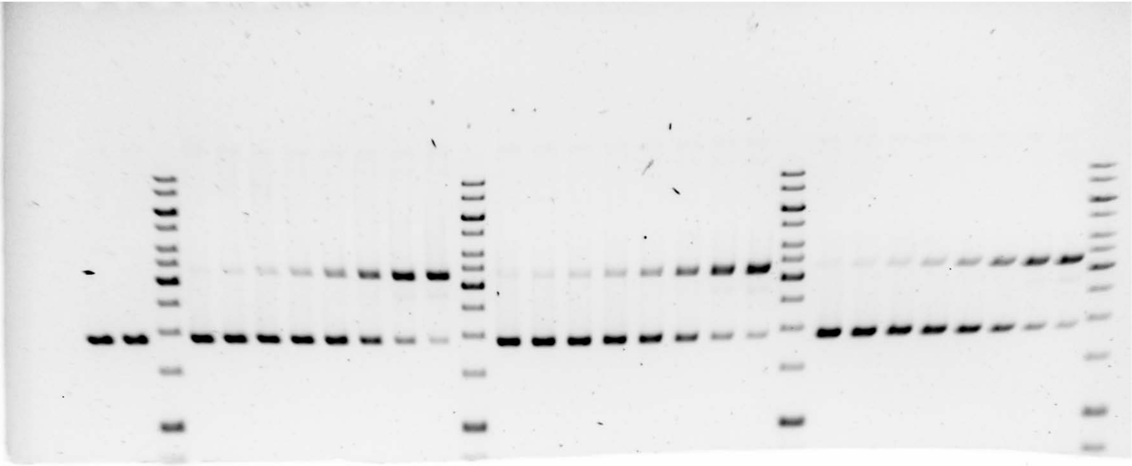

One replicate of WT gene L cleavage with Cas9 bearing the perfect crRNA (left). The samples on the left were used in the gel image shown in S9B Fig. The samples are loaded in the order shown in S9B Fig. The samples on the right are not relevant to this manuscript.

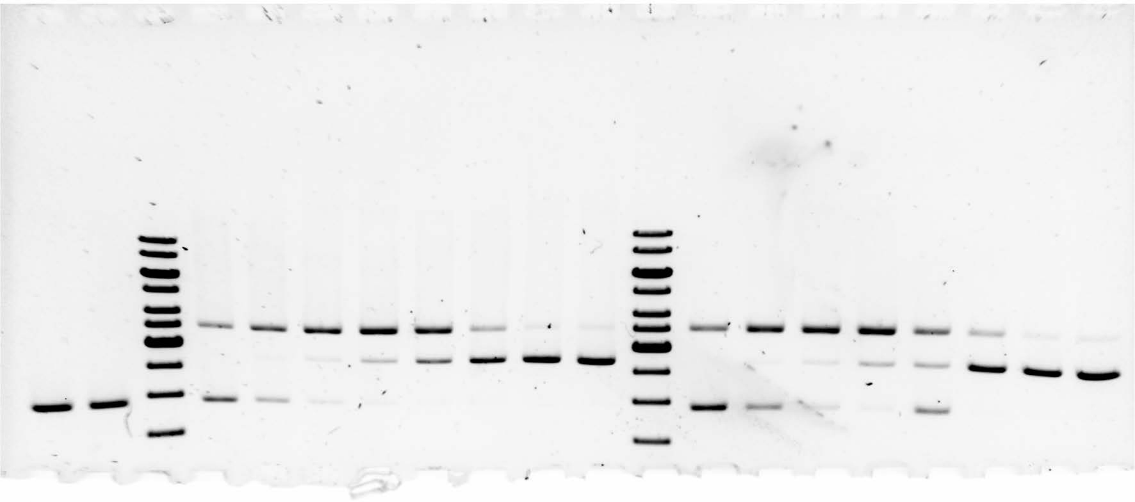

One replicate of WT gene L cleavage with Cas9 bearing the perfect crRNA (left). The samples are loaded in the order shown in S9B Fig. The samples on the right are not relevant to this manuscript.

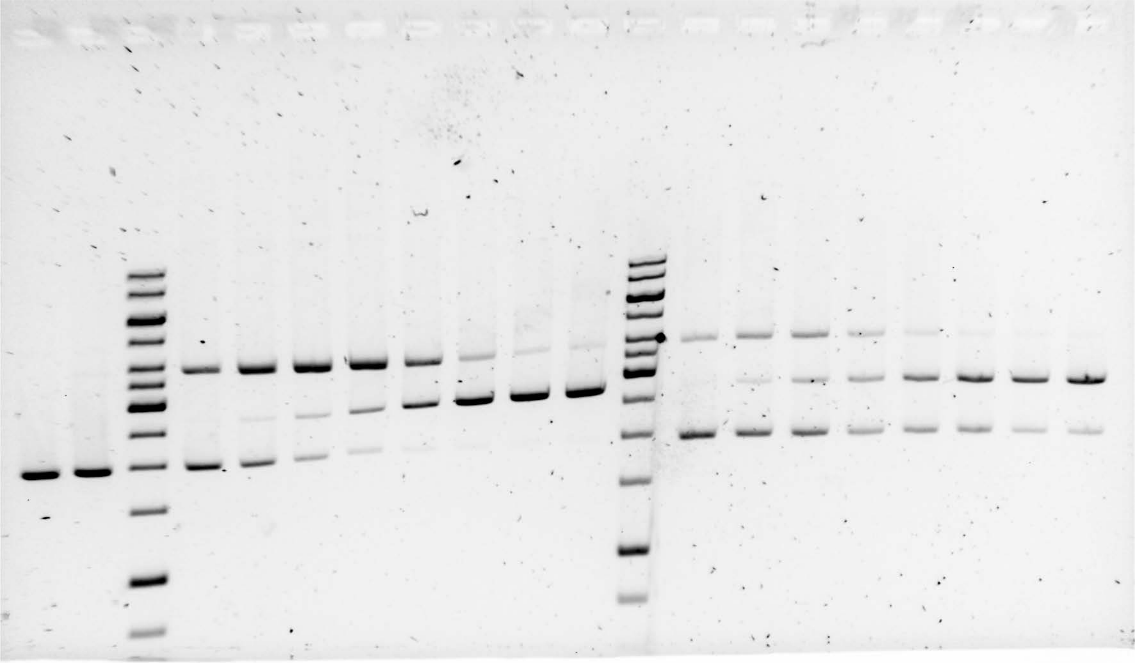

One replicate of WT gene L cleavage with Cas9 bearing the perfect crRNA. The samples are loaded in the order shown in S9B Fig.

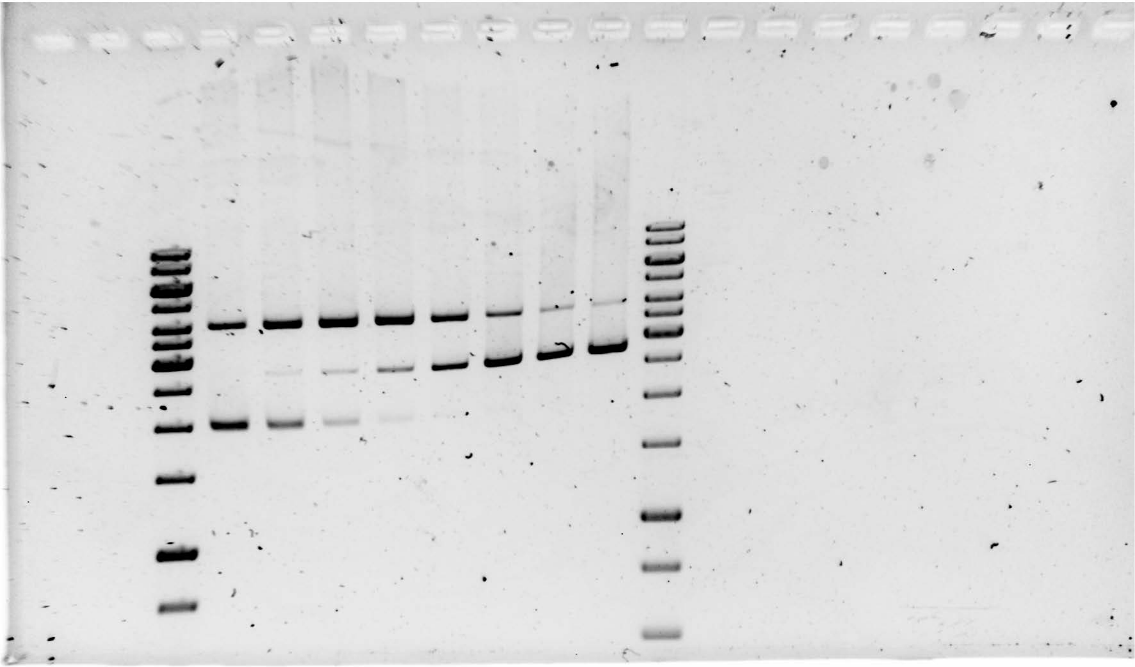

Two replicates of G2T gene L cleavage with Cas9 bearing the perfect crRNA. The samples on the left were used in the gel image in S9B Fig. The samples are loaded in the order shown in S9B Fig.

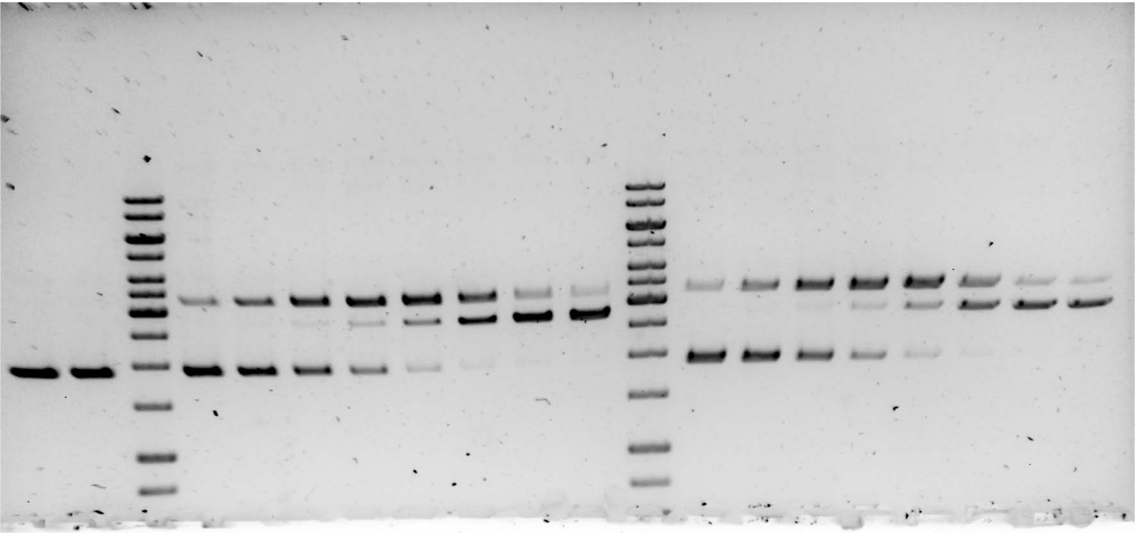

One replicate of G2T gene L cleavage with Cas9 bearing the perfect crRNA (left). The samples are loaded in the order shown in S9B Fig. The samples on the right are not relevant to this manuscript.

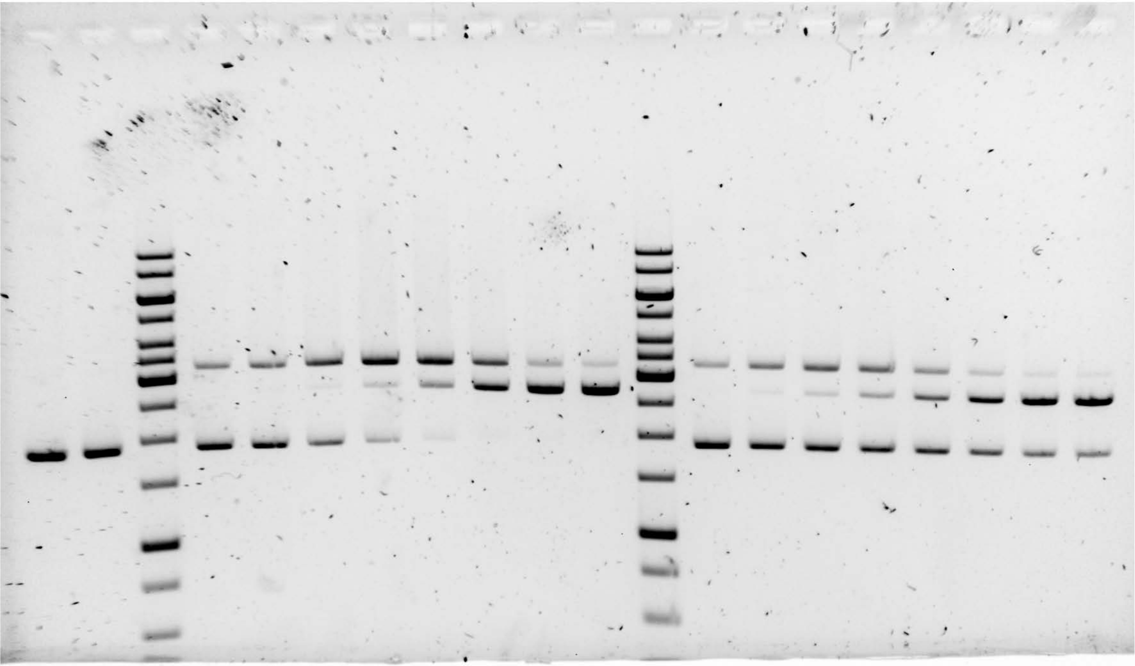

One replicate of A17T gene L cleavage with Cas9 bearing the perfect crRNA (left). The samples on the left were used in the gel image shown in S9B Fig. The samples are loaded in the order shown in S9B Fig. The samples on the right are not relevant to this manuscript.

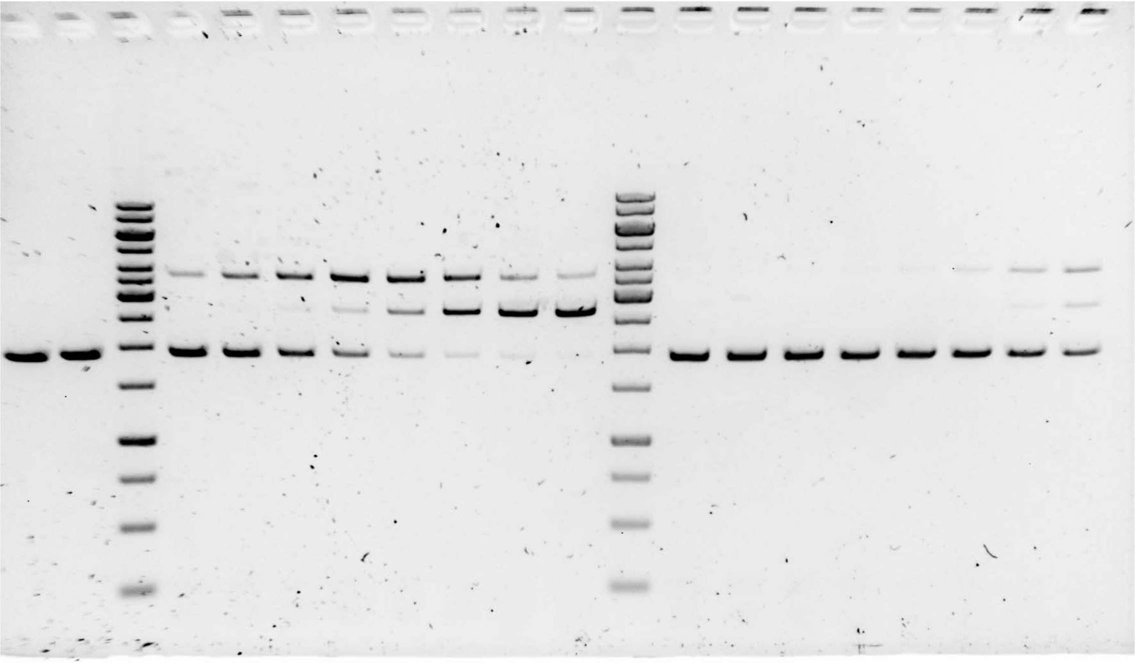

One replicate of A17T gene L cleavage with Cas9 bearing the perfect crRNA (left). The samples are loaded in the order shown in S9B Fig. The samples on the right are not relevant to this manuscript.

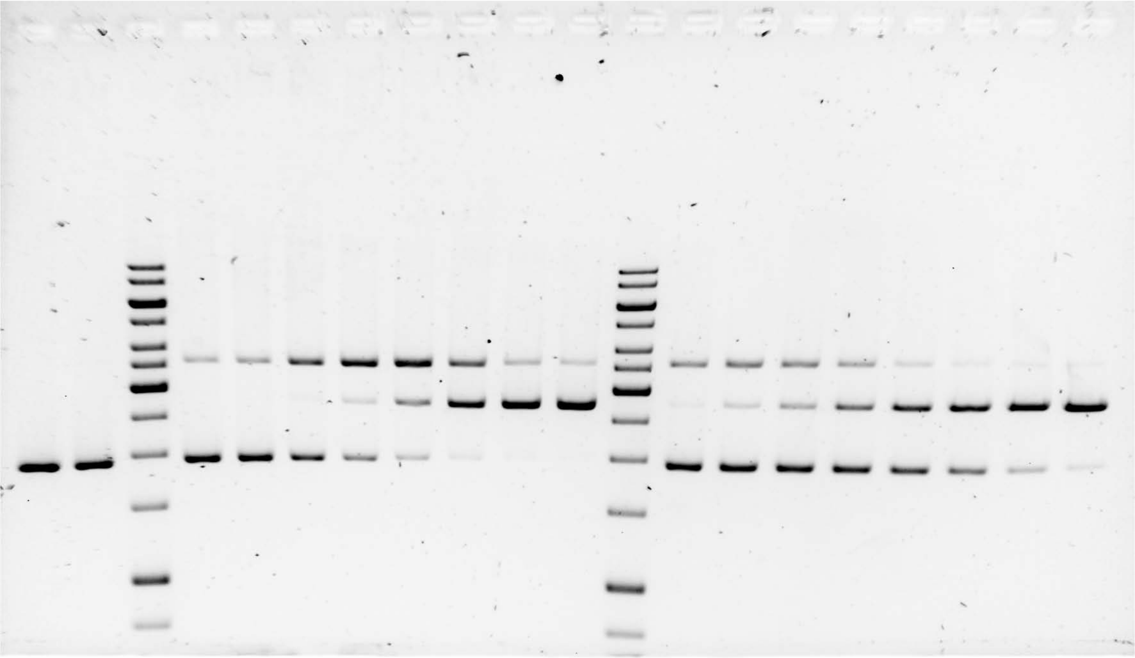

One replicate of A17T gene L cleavage with Cas9 bearing the perfect crRNA (left). The samples are loaded in the order shown in S9B Fig. The samples on the right are not relevant to this manuscript.

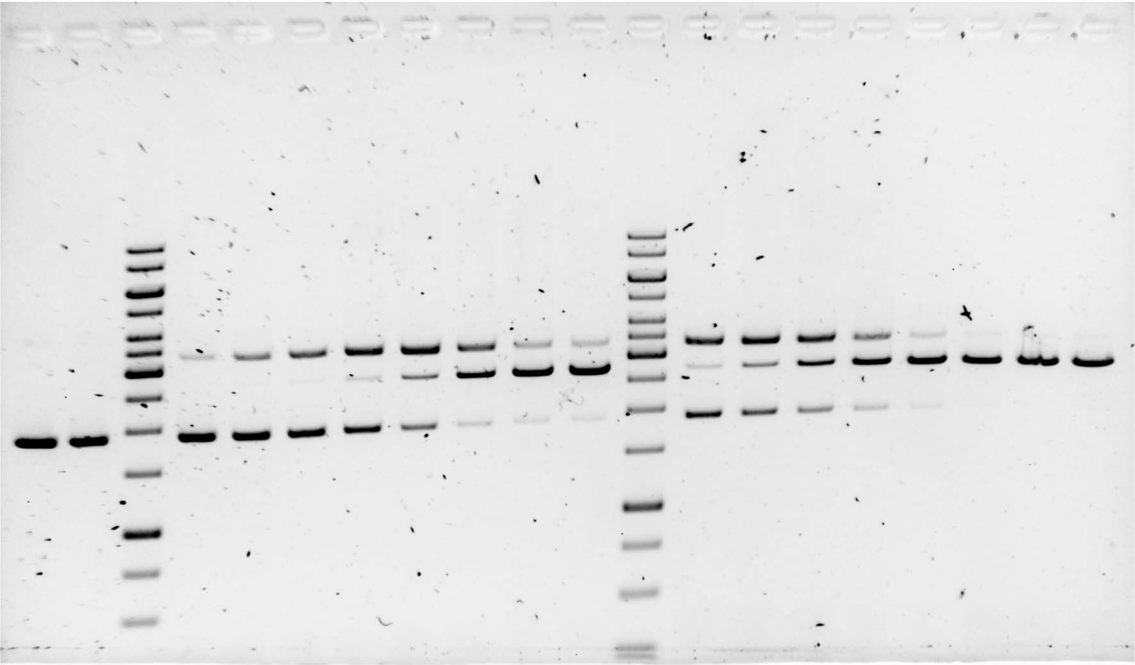

Two replicates of WT gene L cleavage with Cas9 bearing the MM15 crRNA. The samples on the left were used in the gel image in S9B Fig. The samples are loaded in the order shown in S9B Fig.

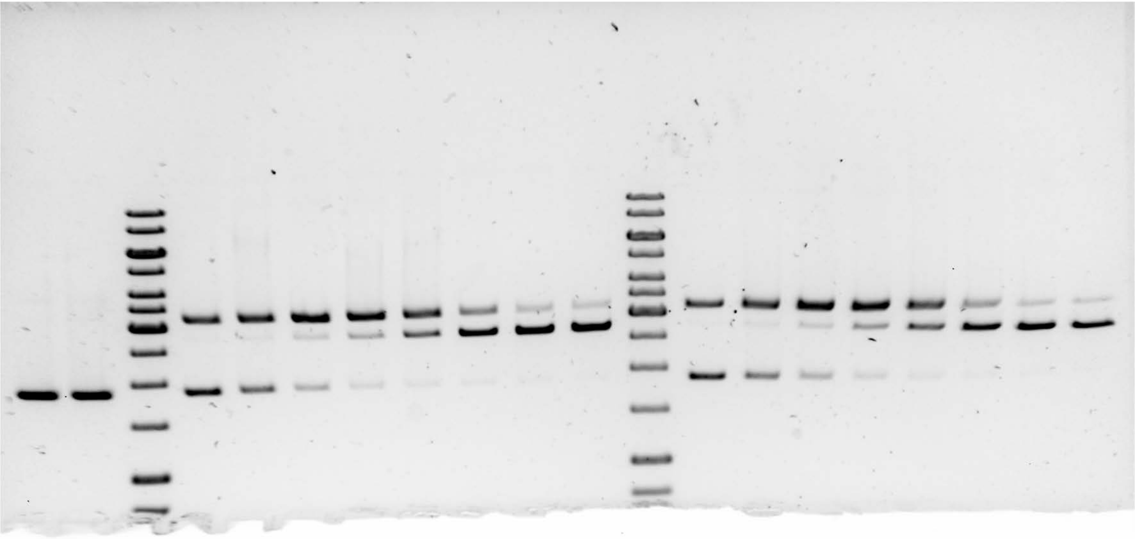

One replicate of WT gene L cleavage with Cas9 bearing the MM15 crRNA (left). The samples are loaded in the order shown in S9B Fig. The samples on the right are not relevant to this manuscript.

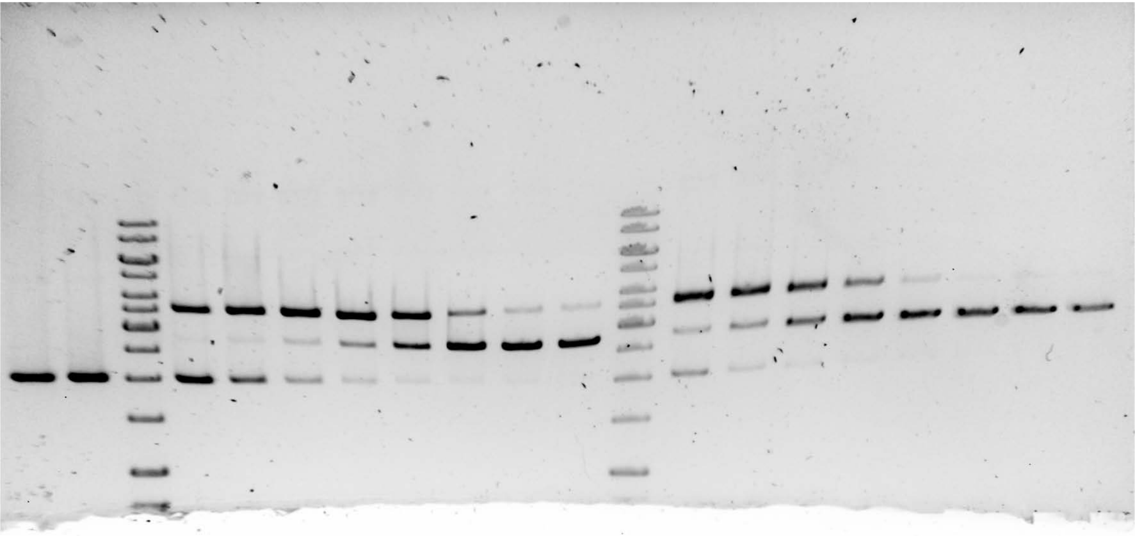

Two replicates of G2T gene L cleavage with Cas9 bearing the MM15 crRNA. The samples on the left were used in the gel image in S9B Fig. The samples are loaded in the order shown in S9B Fig.

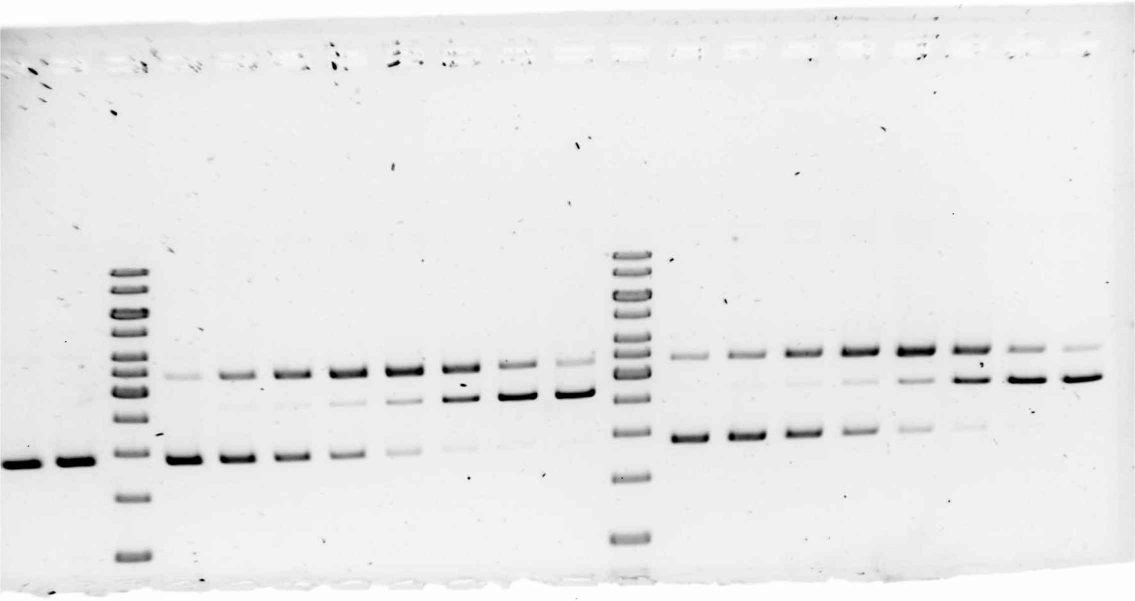

Two replicates of A17T gene L cleavage with Cas9 bearing the MM15 crRNA. The samples on the left were used in the gel image shown in S9B Fig. The samples are loaded in the order shown in S9B Fig.

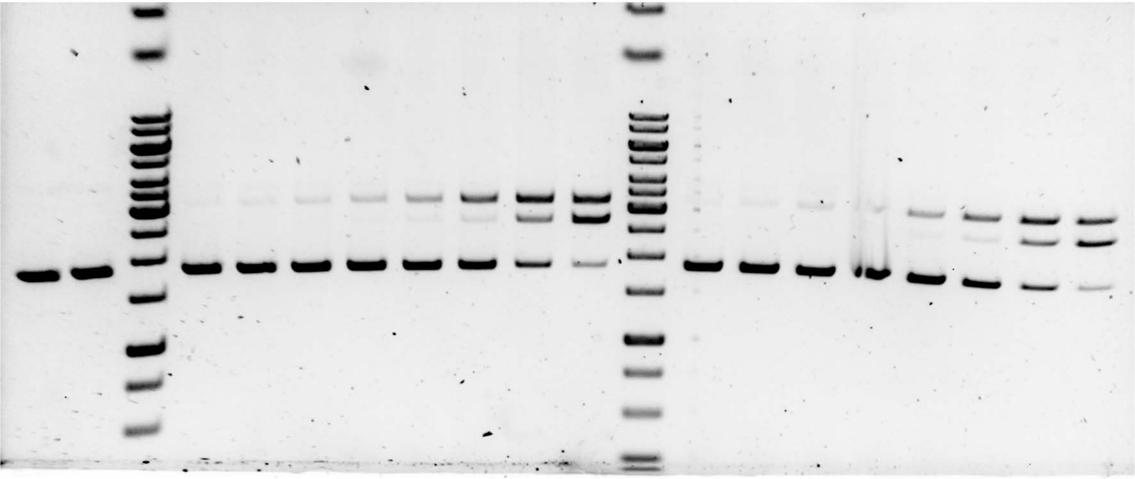

One replicate each of G2T (left) or A17T (right) gene L cleavage with Cas9 bearing the MM15 crRNA. The samples are loaded in the order shown in S9B Fig.

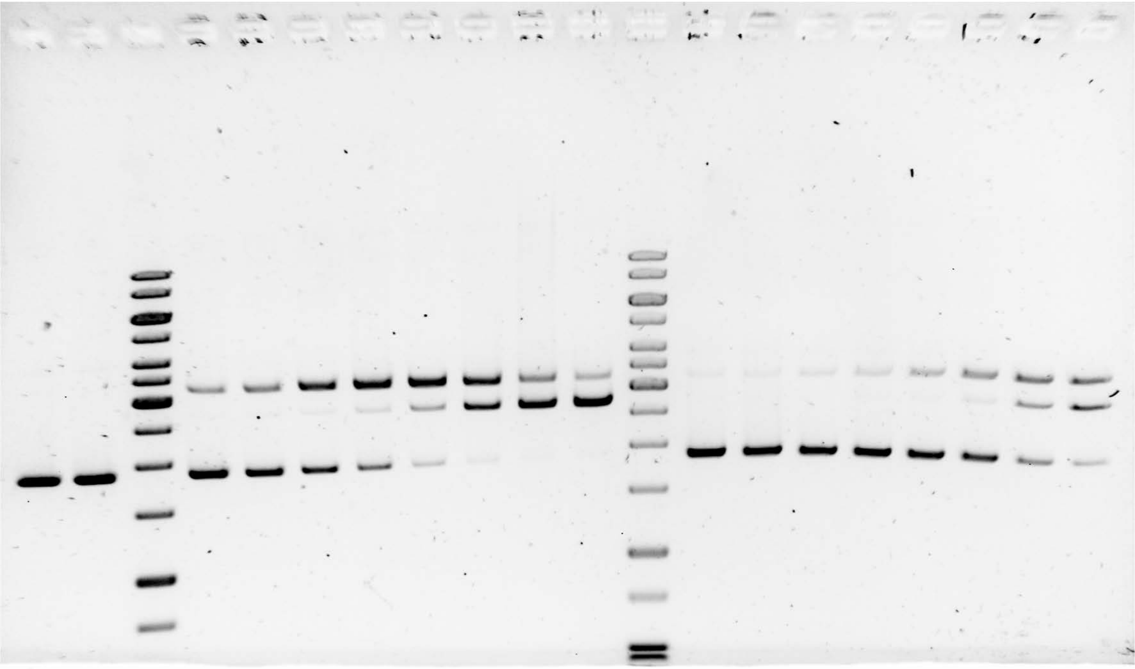

Supplement: S1 Raw Images — Original gel images for all images presented in the Supporting information figures or used for triplicate quantification of Cas12a or Cas9 cleavage rates. All gels were visualized using SyberSafe staining. When relevant, annotations list which samples were used for gel images in S1B, S5A, S7B, S8B or S9B Figs. Samples that were not included in the Supporting information figures contain replicates that were used for quantification of observed rate constants. All samples are ordered as labeled in the relevant Supporting information figure image. (PDF) [file pbio.3002065.s023.pdf]
