# Supplementary material for: CRISPR-Cas effector specificity and cleavage site determine phage escape outcomes
Source: PLoS Biol. 2023 Apr 14;21(4):e3002065. doi: 10.1371/journal.pbio.3002065 (PMC10132644; doi:10.1371/journal.pbio.3002065)
Supplement: Attachment — Submitted filename: ReviewerResponse_submit.pdf [file pbio.3002065.s024.pdf]

*We thank the reviewers for their constructive comments. We have performed several new experiments to address questions about the mechanism of enrichment of phages bearing PAM-distal mutants. We describe these new experiments in the point-by-point response below. The new experiments resulted in several new figures or figure panels and a significant rearrangement of some sections of the manuscript. Changes to the text have been highlighted in yellow in the manuscript uploaded for reviewers. The line numbers referred to below are for that manuscript file. Please note that Supplementary Figures are now labeled S1 Figure, S2 Figure etc. to comply with the journal's formats.*

Reviewer #1: CRISPR-Cas systems use RNA-guided nucleases to interfere against foreign genetic elements, like bacteriophage, in a sequence-specific manner. Because targeting is based on homology between the crRNA and the target sequence, phages frequently escape immunity by evolving mutations in the target site. The impact of these mutations on Cas protein recognition and cleavage depends on their location within the target sequence, PAM and seed mutations confer the greatest resistance to CRISPR interference, while PAM-distal mutations usually have minimal effects. Here the Sashital group discovered that mismatches in all areas of the spacer/target restore the ability of phages to overcome interference by the nuclease Cas12a, even if they do not affect target DNA cleavage in vivo or in vitro. The authors show that these mismatches promote the rise of phage mutants containing additional mismatches in the target region, which more completely evade CRISPR immunity. This is a very thorough paper, with nicely presented data that fully supports the conclusions. I commend the authors for their nice work, and support publication with very minor editorial revisions regarding the points below.

*Thanks to the reviewer for their supportive comments!*

The data in Fig 2C show that the additional phage escape mutations that arise in the context of pre-existing crRNA:target mismatches correlate nicely with the site of the pre-existing mismatch. E.g. seed mismatches promote the accumulation of additional seed and PAM mutations, while PAM-distal mismatches accumulate additional PAM-distal mismatches. To me the observation that the observed escape mutations cluster near the prior mismatch is fascinating, and warrants a bit of additional discussion:

1. Does it not suggest that the additional escape mutations are not pre-existing in the phage population and might be instead introduced during repair after Cas12 targeting? Since PAM and seed mutations are strongly advantageous for the phage, and are sufficient to evade targeting on their own, I would expect these to accrue in all circumstances regardless of the location of the previous mismatch, but this is not what is observed. To me the data support a model in which these escape mutations are actively being introduced.

*This comment is similar to comments from reviewers 2. We agree that there is strong evidence in our data that mutations are actively introduced in the phage. This is especially clear from some of our new data (described below). As a result, we have modified discussion of the mechanisms by which mutants may appear in the population to better account for the strong possibility that mutations arise upon DNA repair following Cas12a cleavage. A new section in the Results entitled "Phage mutations can arise following exposure to Cas12a" starting on line 208 covers much of this discussion. There is also more discussion of this topic in the Discussion sections in the paragraph starting on line 519.*

*We agree that it is surprising that PAM-distal mutants arise when there is a pre-existing mismatch in the PAM-distal region. We previously showed that two PAM-distal mismatches could completely ablate Cas12a cleavage for some of the crRNA/mutant pairs detected in our phage escape experiments (Fig. 3b). We also note that our previous results in S2b Fig. showed that PAM/seed mutants do indeed arise for the*

*PAM-distal mismatched crRNAs when phage infection occurs on solid media. We hypothesize that in liquid cultures, PAM/seed mutants may arise but may be out-competed by PAM-distal mutants that cause more deleterious effects in combination with the pre-existing mismatch. We have now added further discussion of these previous results to lines 284-287.*

*We have also performed new experiments that further test the hypothesis that dual-PAM-distal mismatches may be more deleterious than seed mismatches. To test this, we used mutant phages containing a seed or PAM-distal mutation in the gene L target. On solid media, the seed mutant is far more deleterious than the PAM-distal mutant when infecting* E. coli *expressing the perfect crRNA. In contrast, both mutant phages are readily able to infect* E. coli *expressing the PAM-distal-mismatched crRNA. We further show that the combination of PAM-distal mismatches is more deleterious to in vitro cleavage than the combination of seed and PAM-distal mismatches. Finally, we use a phage competition assay, in which the two mutants were initially mixed at equal titers and used to infect* E. coli *expressing the PAM-distal mismatched crRNA. At low dilutions of the phage, which would mimic the early time points after mutants may arise in the population, the PAM-distal mutant phage readily out-competes the seed mutant phage. In contrast, when using a non-targeting crRNA, the seed mutant appears to be slightly more fit than the PAM-distal mutant phage. Overall these results, which are described in the Results section of the manuscript (lines 287-332) and in Figure 3c-f and S8 Fig., indicate that PAM-distal mutants were selected due to the strongly deleterious effect of two PAM-distal mismatches.*

2. Cas12 is known to cleave ssDNA nonspecifically in response to target binding. Could collateral cleavage at the exposed ssDNA at the mismatch bubble be responsible for the accumulation of escape mutations?

*It is possible that Cas12a non-specific cleavage or target trimming activities may contribute to acquisition of mutations. However, a recent study (PMID: 35670674) suggested that this activity has no impact on anti-phage defense by Cas12a, and it remains unclear whether such collateral cleavage occurs in cells. We therefore believe it is too speculative to mention this possibility as a reason why escape mutations emerge upon Cas12a targeting, especially given the fact that we also observe mutation emergence near the canonical cut site for Cas9 (Fig. 4).*

In the mutation measurements by deep sequencing in Fig 2, it would be interesting to see the number of mutations in the rest of the phage genome (outside the target region). Could the authors present this data if they have it?

*Our deep sequencing was performed only on a PCR amplicon (~110 bp) of the target region. We can therefore only comment on mutations outside of the target region within this 110 bp region of the phage genome. We do not see any substantial accumulation of mutations outside of the target region in these data. We have added sentences describing this to the Results section lines 163-164 and to the figure legend of S3b Fig..*

Line 96: Could the authors specify whether Cas12a was expressed from a plasmid or in the E. coli chromosome, and which promoter was used?

*This information has been added to lines 99-103.*

Line 115: Correlation between cleavage of mismatched target DNA and efficacy in EOP assays - There seems to be a discrepancy between the EOP assay and cleavage rate in the gene J target for some targets.

In particular, the MM8 target is cleaved as well as P in vitro (and better than MM19), but in vivo MM8 is severely impaired in targeting and MM19 is just as susceptible as P.

*Thanks for pointing this out. The reviewer is correct that the gene J MM8 crRNA had no cleavage defect in vitro, but caused a substantial loss of protection when Cas12a was expressed from a weak promoter. This is likely due to escape mutants that are arising due to the presence of the pre-existing mismatch in the crRNA. Indeed, mutant phages were readily detected in cleared spots, as we had previously shown in heat maps in S2b Fig.. These mutations and phage escape may be further accelerated when Cas12a expression is decreased with the weak constitutive promoter leading to more frequent infection in our spot assays. We have added discussion of this to the Results section and changed some wording to better reflect the results:*

Lines 121-125: However, when Cas12a expression was controlled by the weaker promoter, we observed a large loss of protection for the mid-target mismatched cRNA targeting gene J, which had no significant loss of cleavage in our in vitro assay (Fig. 1b,c, S1 Fig.). These results suggest that factors outside of reduced targeting may affect Cas12a-mediated protection at low expression levels.

Lines 146-147: Our initial results showed that crRNA mismatches have less of an effect on solid media than in liquid culture when Cas12a is expressed from a strong promoter.

Lines 481-483: Mid-target and PAM-distal mismatches, however, showed a much more drastic effect in liquid culture than defects observed in vitro or on solid media when Cas12a was expressed from a strong promoter, causing eventual lysis of the bacterial population, sometimes at a rate similar to seed mismatches.

Line 351: "perfect crRNA"

*Thanks, we have corrected this typo.*

Reviewer #2: Schelling et al investigate the impacts of various spacer mismatches on the efficiency of phage targeting by a Type V-A CRISPR-Cas system and the corresponding phage mutations that arise in escapers at the targeted locus. The researchers use Cas12a from Francisella novicida (FnCas12a) expressed in E. coli and phage lambda as a model host-virus system. They demonstrate that mismatches between two representative spacers and their corresponding protospacers in phage lambda cause defects in immunity in a position-specific manner, with seed region mismatches being most detrimental to CRISPR immunity (Fig. 1). The authors go on to characterize phages remaining in culture after Cas12a challenge and show that the position of protospacer mutations in these 'escapers' differ depending upon the position of the mismatched nucleotide (Fig. 2), and that multiple PAM distal mutations can cause loss of immunity by Cas12a (Fig. 3). The multiplicity of infection (MOI) impacts the diversity of phage escaper mutations in the presence of a single mismatch, and combined mismatches are necessary to promote phage escape (Fig. 4). However, two different crRNAs containing mismatches are more effective against phage than individual mismatched crRNAs (Fig. 5). The authors go on to test the impact of protospacer mutations on the diversity of subsequent mutations by isolating individual phage escapers and challenging those with the WT crRNA (Fig. 6). The authors observed that a single mutation naturally-acquired in the seed region is sufficient to afford full protection, while PAM and PAM-distal mutations require the acquisition of additional mutations to allow for phage escape (Fig. 6). Finally, the authors examine the pre-existing mutations in the phage population and found deletions in non-essential

genomic loci which get selected upon Cas12a targeting (Fig. 7).

Overall, the manuscript is well-written and reports on interesting observations relating to the evolutionary dynamics of a phage population when faced with 'imperfect' targeting by Cas12a. The effects of Cas12a on phage evolution is understudied; therefore, these findings are expected to advance our understanding of the biology and impacts of Type V CRISPR-Cas systems. While some of the findings are not surprising (such as the disproportional impact of seed region mutations on Cas12a function, and the presence of pre-existing deletions in nonessential genomic loci in the phage population), there are some compelling observations relating to the compounded inhibitory effect of multiple PAM-distal mutations on Cas12a function, and the diverse mutations that can arise in the presence of mismatches which vary according to the position of the mismatch. However, one major weakness in the study is the absence of definitive evidence explaining how crRNA mismatch position drives mutations in various locations in the protospacer. This and other specific points are listed below in order of importance.

*We thank the reviewer for pointing out the interesting findings in our manuscript and for their constructive comments.*

1. Figure 7: The conclusion that the SNPs in escaper phages following Cas12a 'imperfect' targeting are pre-existing in the population (lines 403-406) is not convincing. The entire paper is focused on the acquisition of SNPs in the essential genes L and J, but when the authors look for pre-existing mutations in the phage population, they only look for deletions in non-essential loci. Is it possible that their origins differ?

*We previously discussed the presence of SNPs in gene J and L, and the difficulties in distinguishing real SNPs from PCR/sequencing error. We provided evidence of single-nucleotide deletions in the wild-type gene J population that were enriched upon Cas12a targeting (S4 Fig. in the current version of the manuscript, previously S9 Fig.). Notably, there were only three single-nucleotide deletions observed in the negative control samples, and two out of three of these also appeared following mutant enrichment. These single-nucleotide deletions in a region upstream of an essential gene, along with the larger deletions observed in non-essential regions, strongly suggest that some mutants that emerge upon phage escape may have been selected from pre-existing mutations in the population.*

*We agree with the reviewer that just because these mutations may be present in the wild-type population, this does not rule out the possibility that they are "re-acquired" through error-prone DNA repair following Cas12a cleavage. We have therefore reduced our emphasis on these results. This section of the Results has been condensed and moved to the first two paragraphs of a new Results section entitled "Phage mutations can arise following exposure to Cas12a" (starting line 208). Figure 7 from the previous version of the manuscript has now been moved to the supplemental materials (S5 Fig.).*

Recent studies have shown that DNA cleavage by Types I and II CRISPR-Cas systems drive mutagenesis in Lambda phage, and this is mainly driven by the phage-encoded repair machinery (Hossain et al, 2021). The conclusion would be more convincing if the authors repeated one of the targeting/mismatch assays in using a mutant phage variant lacking repair machinery and show that the frequency and pattern of mutations in escaper phages remains unchanged.

*We have performed the experiment suggested by the reviewer. We deleted the* red *operon from the lambda-vir genome, and repeated the 8 hour time point to determine whether mutant phages arise when challenged with the perfect or mismatched crRNAs. These results are now included as a heatmap in Figure 2c and in S6 Fig..*

*As observed in Hossain et al., deletion of the* red *operon allowed for stronger CRISPR-mediated defense. We therefore did not observe lysis for many of the cultures tested, and correspondingly did not observe escape mutants. In cultures that lysed, we observed mutants arise in similar locations to the wild-type (WT) phage. For example, for gene L position 19 mismatched crRNA, we observed mutants arising at position 16, which we previously showed is highly deleterious in combination with the position 19 mismatch. These results indicate that the generation of PAM-distal mismatches is not dependent on lambda Red recombination, although it does not rule out the possibility that the mutants arose through an alternative repair mechanism. Indeed, we observe multiple double-mutant phages that emerged when challenged with the gene J position 8 mismatched crRNA. This result strongly suggests that mutants are actively acquired, but also that this acquisition is based on a mechanism that is not dependent on the* red *operon. These new results are discussed on lines 233-259 of the Results and the paragraph starting on line 519 of the Discussion.*

2. Figure 3C: The authors characterize Cas9 phage escapers in the presence of mismatched sgRNAs and were unable to identify PAM-distal mutations using Sanger sequencing. From this observation, the authors conclude that Cas12a may be unique in its susceptibility to PAM-distal mutations (lines 231-236). However, Sanger sequencing is far less sensitive than Illimuna (which was used for the Cas12a escaper characterization). Is it possible that PAM-distal mutants are present following Cas9 targeting, but rare? Can the authors follow-up with Illumina sequencing of Cas9 escapers to show a definitive difference between the two CRISPR types?

*We had originally opted not to use Illumina sequencing of the Cas9 escapers to save resources and because our Cas12a experiments using Sanger sequencing showed results in line with our high-throughput sequencing results. However, we have now performed the experiment requested by the reviewer. The new results do not change our previous conclusions. We do not observe any evidence of PAM-distal mutant emergence for any of the sgRNAs used in the Cas9 experiments. These new results are reported in Fig. 4b-c.*

3. Figure 1: Early in the manuscript, the authors should provide more information about genes K and J. What do they encode? Are they essential for phage survival? The latter is discussed somewhere in the middle of the manuscript, but this information should be introduced along with the assay at the beginning since this greatly affects the types of mutations that can be acquired. Also, why were these specific genes chosen as representatives? Do the authors think that the positional effects of mismatches are generalizable for other spacer sequences when targeting essential genes?

*We have added an explanation for these genes on line 107. These genes were chosen because they both produce essential structural proteins. We believe the positional effects of mismatches are generalizable to other essential genes but might vary depending on the specific target sequence and codon sequence within the essential gene. Notably, we observed the positional effects for both targets, despite the fact that the gene J target was upstream of the coding region while the gene L target was within the coding region. This suggests that similar mutations can arise regardless of position of the target within the gene or intergenic regions. We now mention this in our discussion of mutant emergence (line 506) in the Discussion section.*

4. Figure 2C and D: It is unclear whether the data represents one phage with several point mutations, or many phages, each carrying a smaller subset of the mutations. Can the authors provide more clarification to help guide the reader in the interpretation?

*We thank the reviewer for this suggestion. We have added labels for single point mutations and double-mutants to the legend for this figure and also describe this in the figure legend and in the text (lines 178 and 180).*

5. Figure 5 A, B: The perfect match crRNA control is missing.

*Thank you for the suggestion. We have added these controls (Figures 6b-c of the current manuscript).*

Reviewer #3: Experiments described in this manuscript investigate the effects of CRISPR RNA (crRNA) mismatches on the ability of phages to escape targeting by the Cas12a CRISPR-Cas system of Francisella novicida.

The experimental set-up used here involves heterologous expression of Cas12a and crRNA in E. coli. The crRNA molecules expressed were designed to target phage lambda at two different positions. Experiments involved designing spacers with mismatches at several different positions across the spacer region. Many interesting observations arise from these experiments. In particular, the authors find that even though a mismatch has no effect on the ability of the Cas12 complex to cleave DNA in vitro, phages escaping CRISPR immunity arise much more readily when any mismatch is present. Escaper mutations in phages tend to be close to the site of the mismatch in the crRNA. Interestingly, a similar system testing Cas9 gave a very different result.

The experiments performed here are done rigorously and are clearly described. The results are definitely interesting and warrant publication. My problem with this manuscript with respect to publication in PLoS Biology is that I'm uncertain of the big picture conclusions emerging from this work. While the results are interesting and somewhat unexpected in some instances, it is difficult to draw mechanistic insight from these experiments. Since Cas9 behaved differently, I wonder what the difference is between the Cas12 and Cas9 mechanisms that could cause this.

*We thank the reviewer for their constructive comments. We had previously presented a model in which different mutations emerge from Cas12a and Cas9 due to differences in their specificities. Based on comments from the reviewers, we have further explored this possibility.*

*First, we now present very strong evidence that PAM-distal mutants emerge upon Cas12a targeting with PAM-distal mismatched crRNAs due to the highly deleterious nature of dual PAM-distal mismatches. We described these new experiments in detail to the first comment from Reviewer 1 above. Data presented in Fig. 3c-f now show that Cas12a has a larger defect for cleavage when two PAM-distal mismatches are present than when a seed and PAM-distal mismatch are present, and that this stronger defect causes emergence of PAM-distal mutant phage through selection when a seed and PAM-distal mutant phage are placed in competition with one another.*

*We also attempted the same experiments using Cas9. While we were able to perform similar in vitro cleavage assays (Fig. 4a), we could not perform the competition assay because PAM-distal mutant phages never emerged upon Cas9 targeting. Our in vitro cleavage results indicate that Cas9 actually has a similar cleavage defect (and perhaps worse, although this may be sequence dependent) than Cas12a upon introduction of dual PAM-distal mismatches. Nevertheless, we do not observe any PAM-distal mutations, even when phage populations are sequenced by high-throughput sequencing, as requested by reviewer 2 (Fig. 4b-c, S10b Fig.). These results refute our previous model that differences between Cas12a and Cas9 specificity result in different locations of escape mutations. Importantly, mutations*

*observed for Cas9 all cluster close to the Cas9 cleavage site, while PAM-distal mutations that emerged upon Cas12a targeting are in close proximity to the Cas12a cleavage site. Thus, it is possible that mutations are enriched near the cleavage site due to acquisition of mutations upon Cas effector cleavage and subsequent DNA repair. We now present this model in several locations throughout the paper, including in the title, the abstract (lines 17-19, 23), introduction (lines 89-92, 95), results (section beginning on line 330), and discussion (lines 512-539).*

Is it possible that mismatches could affect the Cas12 non-specific nuclease activity that is induced upon target-binding? Why did the authors focus on Cas12?

*As mentioned above to reviewer 1, we do not believe there is evidence that this type of activity occurs in cells, and it would be speculative for us to comment on this in the text. We also note that it is currently not possible to decouple the non-specific activity observed in vitro from the canonical cleavage activity of Cas12a, e.g. through point mutations in the enzyme active site. We have worked extensively to find mutations that ablate the non-specific nuclease activity but have as of yet been unsuccessful. Thus, testing which activity of Cas12a may contribute to mutant emergence is not possible.*

*Although we have been interested in studying the non-specific nuclease activity of Cas12a in cellular contexts, our main reason for focusing on Cas12a is because its role in natural immune defense is understudied, in our opinion. We have previously extensively studied the specificity of Cas12a in vitro and now sought to extend our findings to determine the effects of Cas12a specificity on phage targeting. This was mentioned as a motivation for this study in both the abstract (lines 6-7) and introduction (75-77). Cas12a (originally Cpf1) was discovered after the genome editing revolution, and little work had been done on characterizing its ability to provide phage defense when we began our study. While other studies on Cas12a-mediated phage defense have been published since the inception of our project, we believe our manuscript underscores the importance of further study of Cas endonucleases as immune effectors, which can reveal new insights into the specificities and mechanism of these effectors.*

The authors should attempt to draw some more general conclusions from this work if it is possible.

*Our new experiments provide stronger conclusions for the mechanism of PAM-distal mutant emergence when Cas12a-crRNA complex bears a PAM-distal mutant emergence. We show that these combinations of mutations are more deleterious than seed mutations, and that PAM-distal mutant phages will emerge in head-to-head competition against seed mutant phages when targeted by a PAM-distal mismatched crRNA. Our new in vitro cleavage results for Cas9 strongly suggest that Cas12a and Cas9 have similar defects for cleavage of targets bearing two PAM-distal mismatches against the crRNA. The differences between Cas12a and Cas9-mediated mutant emergence may be due to cut site differences between the two enzymes. We now present this model as another major conclusion of the study.*

Some minor comments:
Line 107-Authors should briefly discuss the effects of the point mutations in vitro. Are these the expected results. A reader who is unfamiliar with the system may be expecting larger effects.

*We have added more discussion of these results on lines 119-121:*

These results correlated with the cleavage defects measured in vitro for the corresponding mismatched crRNAs, where seed mismatches had stronger defects than other mismatches but still enabled complete cleavage (Fig. 1c, S1 Fig.), consistent with previous results[53,54].

Line 169-Authors need to refer to a figure here.

*The figure reference has been added.*

Figure 4 could be improved by providing schematics of the experiments. These experiments were somewhat complicated.

*We thank the reviewer for the suggestion. We have added schematics for more experiments, including the figure mentioned by the reviewer (now Fig. 5a and 5d) along with Fig. 6a and the new competition experiment performed in Fig. 3f (schematic in Fig. 3e).*
